# Detection of bacterial gene expression elements on Tobacco mosaic virus RNA using cDNA analysis

**DOI:** 10.1371/journal.pone.0358128

**Published:** 2026-09-15

**Authors:** Yoshiyuki Nishimiya

**Affiliations:** Biomanufacturing Process Research Center, National institute of Advanced Industrial Science and Technology (AIST), Sapporo, Japan; Rutgers University/New Brunswick, UNITED STATES OF AMERICA

## Abstract

Tobacco mosaic virus (TMV) is a positive-stranded RNA virus that infects plants. Interestingly, the 5’-untranslated region (UTR) of the TMV RNA genome is recognized and translated by the ribosomes of *Escherichia coli* in a Shine–Dalgarno (SD) sequence-independent manner. This study aimed at investigation of the bacterial recognition modules that control gene expression within the TMV RNA genome. To this end, the 5’-end-complete cDNA of the TMV RNA and several 5’-end-truncated cDNA mutants, in which the movement protein-encoding gene and its downstream region were replaced with a DNA sequence encoding a green fluorescent protein, i.e., monomeric Umikinoko-Green (mUkG1), were constructed. Surprisingly, mUkG1 fluorescence was observed in *E. coli* transformants harboring the cloned cDNAs, although they were inserted into a vector lacking a promoter. Analysis of the 5’-end-truncated cDNA mutants and promoter prediction suggested that an *E. coli*-specific promoter might be located 2.1 kb upstream of the initiation codon for mUkG1. Furthermore, Western blotting analysis and conversion of the initiation codon ATG to AGT indicated that the translation of mUkG1 started from the correct initiation codon. These results imply that *E. coli* ribosomes correctly recognize the initiation codon on the mRNA, irrespective of the overly long 5’-UTR. To the best of our knowledge, this report is the first to reveal a recognizable bacterial module hidden within the TMV RNA genome through cDNA construction.

## Introduction

Viruses lack a protein translation machinery and instead utilize their hosts’ cellular machinery to translate their mRNA and produce viral proteins, including structural capsid proteins and nonstructural proteins, such as replicases and RNA-silencing suppressor proteins. Viral genomes are composed of either DNA or RNA in the double-stranded (ds) or single-stranded (ss) forms [1]. The virome of eukaryotes mostly contains RNA viruses, especially positive-stranded RNA (+RNA) viruses [2]. In eukaryotic cells, the genetic material is enclosed within the nuclear envelope. Therefore, viruses infecting the cytoplasm of host cells cannot readily access the host’s DNA replication and transcription machinery [3,4]. Negative-stranded RNA (−RNA) and dsRNA viruses produce mRNAs in the host cytoplasm using their RNA genomes as templates, thereby initiating viral replication. Therefore, these viruses package their transcription and replication machineries into their virions [1,5,6]. Conversely, the genomes of +RNA viruses can function directly as mRNAs in the host cytoplasm, which might explain why RNA viruses, especially +RNA viruses, are predominant in the virome of eukaryotes [2].

Tobacco mosaic virus (TMV) is a + RNA virus belonging to the genus *Tobamovirus* and the family *Virgaviridae*, which can infect solanaceous plants. Its complete RNA genome sequence was reported in 1982 [7]. This genome encodes four viral proteins: a 126-kDa protein [comprising the methyltransferase (MT) domain, the intervening region (IR), and the RNA helicase (Hel) domains], the read-through 183-kDa replicases [comprising the 126-kDa protein and RNA-dependent RNA polymerase (RdRp) domains], the movement protein (MP), and the capsid protein (CP) [7–9]. The 3’-end of the gene encoding the 183-kDa protein serves as the subgenomic promoter (Sgp) and the N-terminal region for MP, while the 3’-end of the MP gene acts as the Sgp for CP [10]. The 5’-untranslated region (5’-UTR) of the TMV RNA genome, also known as the omega (Ω) sequence, contains a poly-CAA region, which functions as a translation enhancer in plants [11,12]. Conversely, the 3’-UTR can fold into a tRNA-like structure that contributes to efficient genome replication [13,14]. Interestingly, the TMV genome has been shown to replicate in non-native eukaryotic hosts, including *Saccharomyces cerevisiae* [15]. Moreover, reports have shown SD sequence-independent translation of the genomes of RNA viruses, including TMV, in bacteria, such as *Escherichia coli* [16,17]. The 5’-UTR sequences of TMV and Alfalfa mosaic virus RNA4 can be recognized with high specificity and translated by *E. coli* ribosomes. The ribosomal protein S1 in *E. coli* plays a critical role in ensuring accurate translation of these viral sequences [17,18]. While the heterologous translation in bacteria has been investigated, the genetic traces of gene expression elements in plant viral RNAs have remained largely unexplored.

Here, we generated the cDNA sequence of a modified TMV genome, comprising the 5’-UTR, the 183-kDa replicase-encoding region (including the Sgp for MP), and a monomeric Umikinoko-Green (mUkG1) gene (instead of the MP-encoding gene). The Sgp for CP, the CP-encoding gene, and the 3’-UTR were deleted. This cDNA sequence was cloned into a *lac* promoter-deleted pUC19 vector. Surprisingly, *E. coli* transformants harboring the constructs exhibited fluorescence, indicating that mUkG1 is expressed, despite the absence of a promoter and an SD sequence for the mUkG1 gene. In this study, to elucidate the elements that contribute to mUkG1 expression, a deletion analysis from the 5’-end of the modified TMV cDNA and promoter prediction were performed. These analyses suggest that the gene expression elements for mUkG1 are located in the TMV-Hel domain-encoding region, which is far away from the mUkG1-encoding DNA. Furthermore, a comparison with a highly homologous Tomato Mosaic virus (ToMV)-Hel indicated that a sequence resembling a bacterial promoter was located upstream of the gene encoding the RecA-like domain of TMV-Hel. These observations imply a genetic trace of gene expression elements in the TMV genome RNA, which might have been shared by an ancestor of +RNA viruses. Therefore, these findings may be significant for understanding the evolutionary scenarios for RNA viruses in the eukaryotic virome.

## Materials and Methods

### Recombinant strains and growth conditions used for vector construction and mUkG1 expression

The *E. coli* HST08 strain (TaKaRa Bio, Japan) was used for vector construction. The *E. coli* HST08 and KRX strains (Promega, USA) were used for analyzing the expression of mUkG1. *E. coli* cells were grown in Luria-Bertani (LB) medium containing 100 µg/mL ampicillin for pUC-TMx-G and 50 µg/mL kanamycin for pT7-TMx-G at 32 °C. For *in vivo* homologous recombination of DNA fragments, the *Saccharomyces cerevisiae* BY4743 (*MATa/α his3Δ1/his3Δ1 leu2Δ0/leu2Δ0 LYS2/lys2Δ0 met15Δ0/MET15 ura3Δ0/ura3Δ0*) strain (Open Biosystems, USA) was used. The *S. cerevisiae* cells harboring vectors containing the *ura3* gene were grown in synthetic defined medium without uracil (Merck, Germany) at 30 °C.

### Vector construction

**Fig 1:**
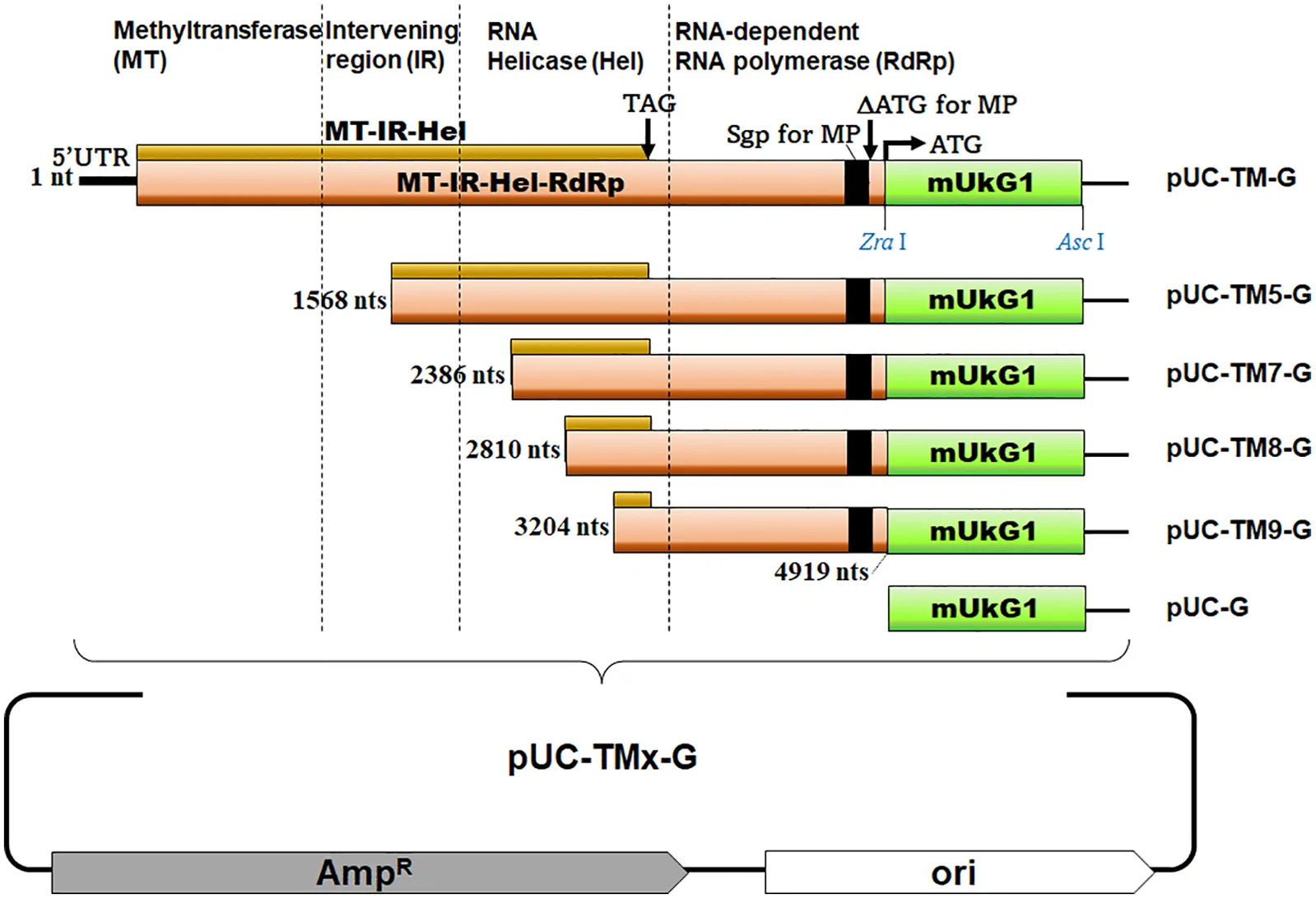
Construction of the pUC-TMx-G vectors. The fragments containing the gene encoding the 5’-untranslated, methyltransferase, intervening, helicase, and RNA-dependent RNA polymerase (RdRp) regions of TMV and mUkG1 were cloned between the PvuII and PvuII sites of the pUC19 vector. The gene encoding RdRp contained the subgenomic promoter for the movement protein (MP), whose initiation codon was deleted via synonymous substitution. The bent arrow indicates the initiation codon for mUkG1.

All DNA fragments and PCR primers used are shown in S1 and S2 Tables. PCR amplifications were performed using the Q5 high-fidelity DNA polymerase (New England Biolabs, USA). Fourteen DNA fragments (TM1, TM2, TM3, TM4, TM5, TM6, TM7, TM8, TM9, TM10, TM11, TM12, TM13–14, and TM15) of the TMV gene and the mUkG1-encoding DNA were synthesized by AZENTA (Burlington, USA). To construct the cDNA for the modified TMV genome, overlap extension PCRs were first performed for each fragment pair (TM1/TM2, TM3/TM4, TM5/TM6, TM7/TM8, TM9/TM10, and TM11/TM12) using the respective primer set [TMx-F and TM(x + 1)-R], generating six extended fragments. These fragments, TM13–14 and TM15, along with the *Bam*HI/*Xba*I-digested pCLY vector (ATTO, Japan), were introduced into *S. cerevisiae* BY4743 using the Frozen-EZ Yeast Transformation II Kit (ZYMO RESEARCH, USA). This resulted in the introduction of the modified TMV cDNA into pCLY via *in vivo* homologous recombination. The transformants were spread on agar plates containing synthetic defined medium without uracil and incubated for 2 days at 30 °C. The colonies formed were harvested and cultured in synthetic defined medium without uracil at 30 °C. The prepared constructs (denoted as pCLY-TM) were purified from the transformants using Zymoprep Yeast Plasmid Miniprep Kits (ZYMO RESEARCH, USA), amplified in the *E. coli* strain HST08, and recovered for subsequent vector construction. The pCLY-TM vector and the mUkG1-encoding DNA were digested with *Zra*I/*Asc*I and *Asc*I, respectively, and ligated (S1 Fig A). The ligated vector (denoted as pCLY-TM-G) was used as a PCR template to construct the deletion mutants from the 5’-end of the TMV gene and the promoter-less mUkG1 gene. Six PCR fragments were amplified using TM1-F, TM5-F, TM7-F, TM8-F, TM9-F, and mUkG1-F as forward primers and TM14-R as the reverse primer. These PCR fragments were digested with *Avr*II and then ligated to the *Sma*I/*Xba*I-digested pUC19 vector (denoted as pUC19-TMx-G and pUC19-G in S1 Fig B). To delete the *lac*Z promoter from pUC19, PCR was performed using pUC19-TMx-G and pUC19-G as the templates and the *Pvu*II-forward/reverse primer set. The PCR amplicons were ligated to the *Pvu*II-digested pUC19 vector using the In-Fusion cloning kit (TaKaRa Bio, Japan) (S1 Fig C). The details of the completed pUC-TMx-G vectors are provided in Fig 1. The pUC-TMx-G vectors were used as PCR templates to construct the T7 promoter-inducible vectors (pT7-TMx-G). Then, these PCR products were cloned into a vector based on the pET26b backbone. The promoter sequences are shown in S3 Table. The sequences of the generated TMV and mutant cDNAs were confirmed by Sanger sequencing.

### Expression analysis of mUkG1

Each *E. coli* HST08 colony harboring pUC-TM-G, its deletion mutant vectors, or pUC19 (as a negative control) was inoculated into 2 mL of LB medium containing ampicillin and pre-cultivated overnight at 32 °C. Then, 20 µL of the overnight culture was transferred into tubes containing 2 mL of freshly prepared LB medium with ampicillin and incubated at 145 rpm and 32 °C for 48 h. During the 48-h incubation, 100 µL of these cultures were transferred into each well of a 96-well microplate at different time points (4, 8, 12, 24, 36, and 48 h) to measure OD_600_ and fluorescence intensity using a multimode microplate reader (Spark; TECAN, Switzerland). The fluorescence was normalized to the OD_600_ values (fluorescence/OD_600_). To estimate the background autofluorescence, the fluorescence of *E. coli* harboring pUC19 was also measured. For the KRX strain, 10 µL of the overnight culture was transferred into each well of a 96-well plate containing 1 mL of freshly prepared LB medium with kanamycin. The plates were incubated at 900 rpm and 32 °C. After 4 h, mRNA transcription was induced by adding 5 µL of 20% rhamnose for the T7 promoter. OD_600_ and fluorescence intensity values were measured after an additional 6 h of culture. Furthermore, the *E. coli* cells harboring pUC-TMx-G were cultivated in 2 mL of LB medium for 24 h at 32 °C and collected by centrifugation for 5 min at 5,000 × *g*. The fluorescence of *E. coli* pellets was observed using a fluorescent excitation light-emitting diode (LED) source (VariRays I; ATTO, Japan).

The expression levels of mUkG1 were analysed using sodium dodecyl sulphate–polyacrylamide gel electrophoresis (SDS-PAGE) and Western blotting. For pUC-TM-G, pUC-TM5-G, pUC-TM7-G, and pUC-TM9-G, a C-terminal hexahistidine tag (6×His-tag) was conjugated to mUkG1 (denoted as pUC-TM-GH, pUC-TM5-GH, pUC-TM7-GH, and pUC-TM9-GH, respectively). After 24 h of culture, *E. coli* cell pellets harboring these vectors were lysed using the BugBuster Master Mix protein extraction reagent (Merck, USA). After normalizing total cellular protein content, the proteins were analysed together with a positive control, i.e., 6×-His-tagged mUkG1 prepared from the BL21 (DE3) strain. For Western blotting, the proteins separated by SDS-PAGE were transferred to polyvinylidene fluoride (PVDF) membranes and then detected using an anti-His-tag mouse monoclonal antibody (MBL, Japan) and a horseradish-peroxidase-conjugated anti-mouse IgG (H + L) goat polyclonal Fab (MBL, Japan) with Clarity Western ECL Substrate (BIO-RAD, USA).

### Rapid amplification of cDNA 5’-end (5’-RACE)

To prepare total RNA, 270 µL of the *E. coli* culture, harvested after 24 h, was mixed with 540 µL of RNA Protect Bacteria Reagent (Qiagen, Germany). After incubation for 5 min, the cell suspensions were centrifuged for 10 min at 5,000 × *g*. Then, the pellets were resuspended in 200 µL of lysis buffer (30 mM Tris-HCl, pH 8.0, 1 mM EDTA, and 15 mg/mL lysozyme) containing proteinase K. After incubation for 10 min, 700 µL of buffer RLT (Qiagen, Germany) containing 40 mM dithiothreitol (DTT) was added to the lysed pellets. Subsequently, total RNA was purified from these pellets using an RNeasy Mini kit (Qiagen, Germany) according to the manufacturer’s protocol. The prepared RNAs were treated with RNase-free DNase I (New England Biolabs, USA) for 1 h at 37 °C. Then, DNase I was heat-denatured for 10 min at 75 °C after the addition of 5mM EDTA. The DNase-treated RNAs were finally purified using the Monarch RNA Cleanup kit (New England Biolabs, USA).

5’-RACE was performed with 5’-Full RACE Core Set (TaKaRa Bio, Japan) according to the manufacturer’s protocol. First-strand cDNAs were synthesized using 2.5 µg of total RNA as the template, 200 pmol of 5’-end-phosphorylated primers for reverse transcription (S2 Table), and 5 U of AMV RT XL for 10 min at 30 °C. This was followed by elongation for 60 min at 50 °C and denaturation for 2 min at 80 °C. Then, the hybrid RNA was degraded using RNase H for 60 min at 30 °C. The single-stranded cDNA was collected from the mixture by ethanol precipitation. Next, these single-stranded cDNAs were circularized or concatenated using T4 RNA ligase for 18 h at 15 °C.

The first PCR was performed using KOD FX Neo (TOYOBO, Japan) with the 1^st^ PCR primer sets and a 10-fold dilution of circularized cDNAs or concatemers as templates. The conditions for the 1^st^ PCR were as follows: one cycle of denaturation for 2 min at 94 °C, 30 cycles of denaturation for 10 s at 98 °C, annealing for 30 s at 65 °C, and elongation for 1 min at 68 °C. The 1^st^ PCR products were diluted 10 or 100-fold and then used as templates for nested PCR (2^nd^ PCR). The conditions for the nested PCR using Taq polymerase (New England Biolabs, USA) and 2^nd^ PCR primer sets were as follows: one cycle of denaturation for 30 s at 95 °C, and 30 cycles of denaturation for 30 s at 95 °C, annealing for 15 s at 49 °C, and elongation for 2 min at 68 °C. The 2^nd^ PCR products were TA-cloned into the pMD19 vector (TaKaRa Bio, Japan). The 5’-ends of cloned cDNAs were determined by Sanger sequencing using BigDye Terminator Cycle Sequence Kit (Thermo Fisher Scientific, USA).

### Promoter prediction

The promoter regions were predicted by the bacterial sigma-70 promoter recognition program (BPROM) online software (http://www.softberry.com/).

### Reverse transcription-quantitative PCR (RT-qPCR)

For the preparation of total RNAs, 270 μL of E. coli cultures after 24 h of culture were mixed with 540 μL of the RNA protect Bacteria Reagent (Qiagen, Germany). After incubation for 5 min, the pellets were recovered by centrifugation for 10 min at 5,000 × g and resuspended in 200 μL of lysis buffer (30 mM Tris-HCl pH 8.0, 1 mM EDTA, and 15 mg/mL lysozyme) containing proteinase K. Seven hundred microliters of buffer RLT (Qiagen, Germany) containing 40 mM DTT were added to the lysed pellets after incubation for 10 min, followed by the purification of total RNAs from the RLT-treated pellets using an RNeasy mini kit (Qiagen, Germany) according to the manufacturer’s protocol. The prepared RNAs were treated with RNase-free DNase I (NEB, USA) for 1 h at 37 °C, and DNase I was heat-denatured for 10 min at 75 °C after the addition of EDTA (final concentration, 5 mM). Next, reverse transcription of DNase-treated RNAs was performed using the High Capacity RNA-to-cDNA Kit (Thermo Fisher Scientific, USA) for 1 h at 37 °C, followed by denaturation for 5 min at 95 °C. RT-qPCR was performed using the prepared cDNAs and the Power SYBR Green PCR Master Mix on a QuantStudio 3 (Thermo Fisher Scientific, USA). The reaction conditions were as follows: incubation at 50 °C for 2 min and 95 °C for 10 min, followed by 45 cycles of 95 °C for 15 s and 60 °C for 1 min. The relative expression of the modified TMV cDNAs was calculated via the ΔΔCt method with pUC19-ΔSD-G as a reference using the Design and Analysis software ver. 1.4 software (Thermo Fisher Scientific, USA). The *E. coli* siroheme synthase (*cysG*) was used as a housekeeping gene for normalization.

### Statistical analysis

All experimental analyses were performed in biological triplicate. The Welch t test was used to determine the significance of the difference between the means of two groups.

## Results

### Design of modified TMV cDNAs

To prepare the vectors for the modified TMV cDNAs, the regions consisting of the MP gene, the Sgp for CP, the CP-coding genes, and the 3’-UTR of the TMV genome were replaced with cloning sites (*Zra*I/*Asc*I/*Avr*II/*Not*I). Then, the mUkG1-encoding gene was introduced using the *Zra*I and *Asc*I sites (Fig 1). The DNA fragments used for constructing the vectors are listed in S1 Table. More specifically, the initiation codon of MP in the 3’-end of the RdRp-domain-encoding gene was deleted using synonymous substitution (G*ATG*GC to G*ACG*GC), and a *Zra*I site was inserted just after the termination codon of the RdRp domain. Four truncated mutants, lacking the region from the 5’-UTR to the N-terminal of the IR, or the N-terminal side of the Hel domain of the TMV replicase, were designed. One of them, pUC-TM5-G, retained the Hel domain but lacked the first half of the IR. The remaining three mutants (pUC-TM7-G, pUC-TM8-G, and pUC-TM9-G) gradually lost the Hel domain (Fig 1). Specifically, pUC-TM7-G and pUC-TM8-G lost the N-terminal region and the first half of the Hel domain, respectively, and pUC-TM9-G included only the C-terminal region of the Hel domain. pUC-G was constructed as the negative control. For all vectors, the modified TMV genes and the mUkG1 gene were introduced into the lac promoter-deleted pUC19 vector in the opposite direction of the ampicillin resistance gene cassette (Amp^R^) and replication origin.

### Detection of fluorescence intensity in *E. coli* harboring pUC-TMx-G

**Fig 2:**
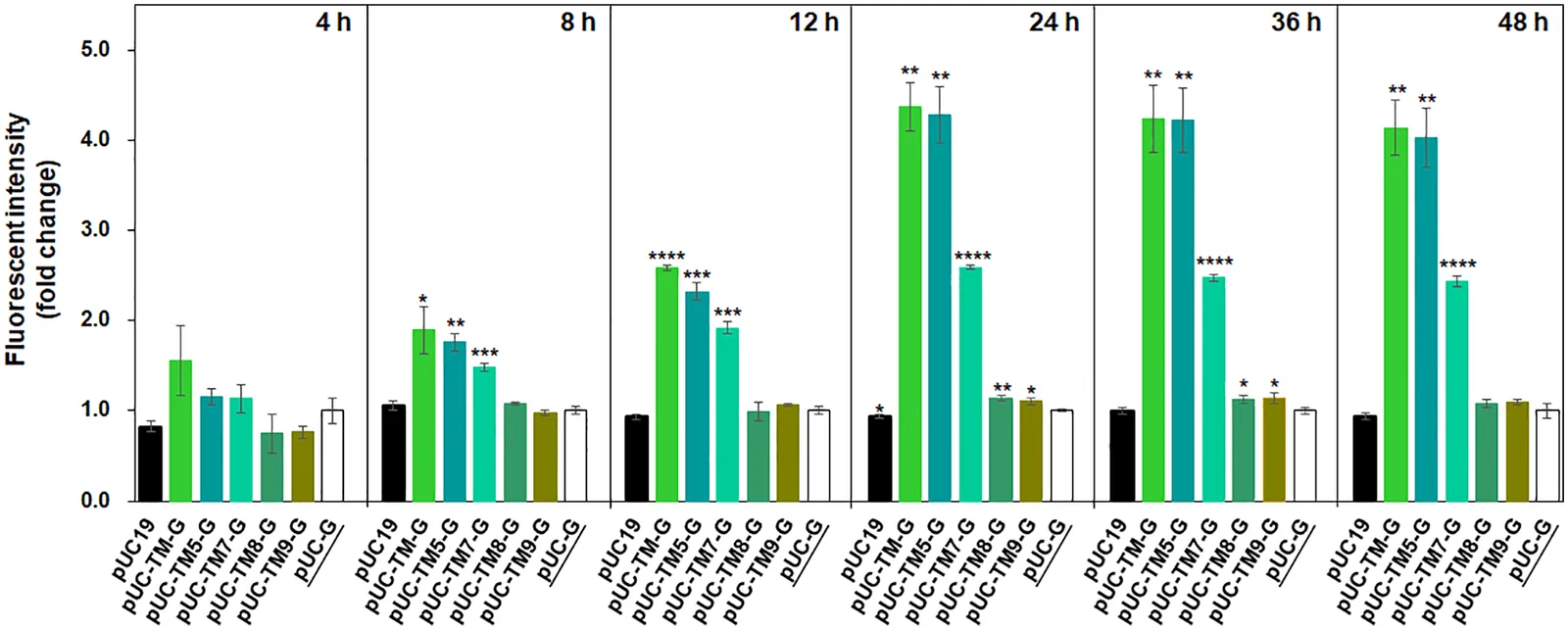
Fluorescence intensity analysis of *E. coli* harboring the pUC-TMx-G vectors. Fold changes in fluorescence intensities for each *E. coli* culture over the control, i.e., pUC-G (white bar with underlined label), were calculated at different cultivation time points (4, 8, 12, 24, 36, and 48 h), as shown. Data are presented as the mean ± standard deviation of three biological replicates. Significant differences from pUC-G are indicated by *P < 0.05, **P < 0.01, ***P < 0.001, and ****P < 0.0001.

**Fig 3:**
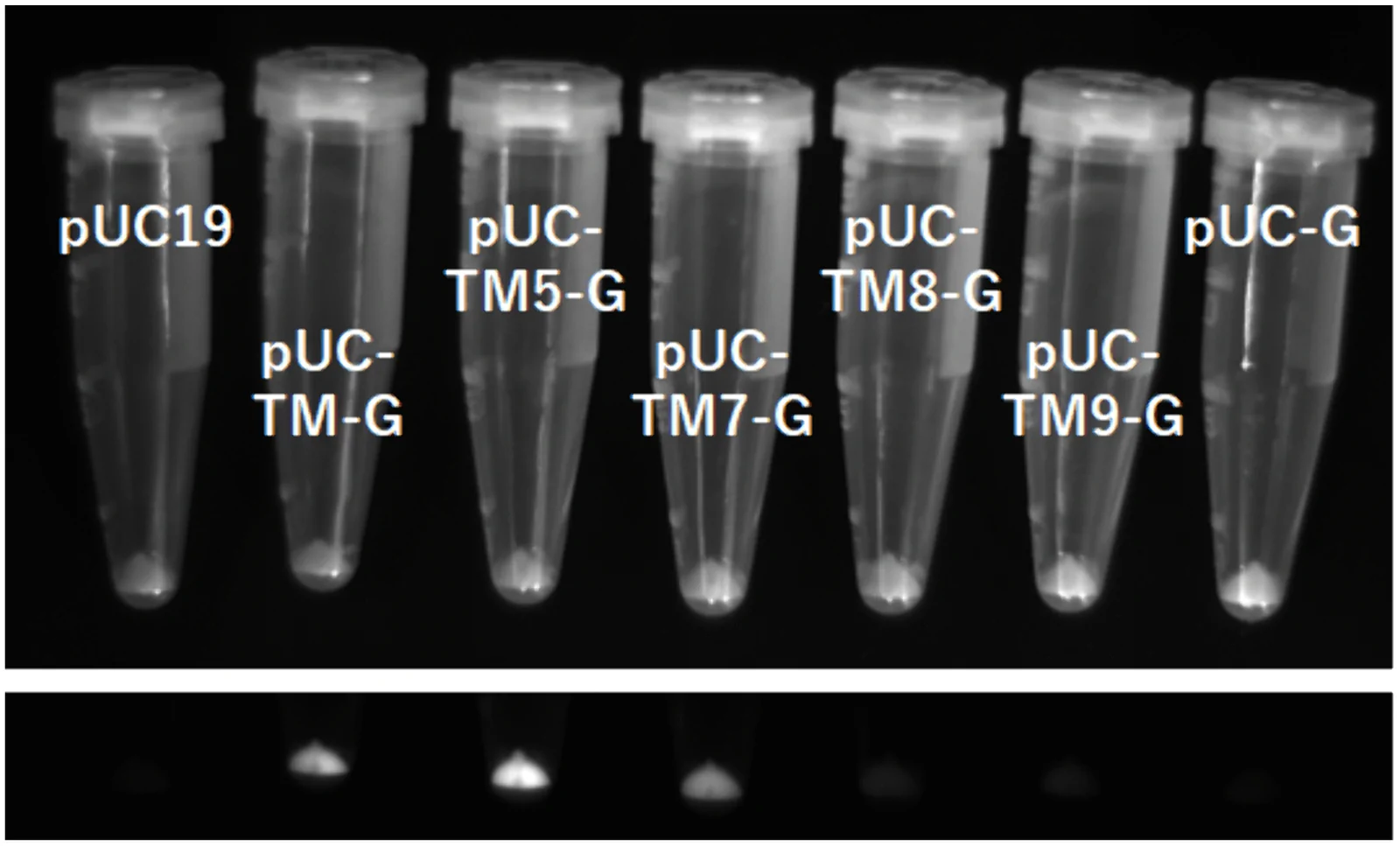
Expression analysis of mUkG1 in *E. coli* harboring the pUC-TMx-G vectors. Photographs of cell pellets before (up) and after (down) exposure to a fluorescent excitation LED light source after cultivation for 24 h.

As mUkG1 expression can be quantified based on fluorescent intensities, the fluorescence intensities of the *E. coli* HST08 cells harboring pUC-TMx-Gs were measured at different time points (4, 8, 12, 24, 36, and 48 h) (Fig 2). There was no difference in the fluorescence intensities between pUC-G and the pUC19 mock vector, indicating that mUkG1 was not expressed by pUC-G and an autofluorescence in *E. coli*. In contrast, pUC-TM-G, pUC-TM5-G, and pUC-TM7-G exhibited significantly higher fluorescence intensities at 8, 12, 24, 36, and 48 h. Notably, the fluorescence intensity of cells harboring pUC-TM-G and pUC-TM5-G was more than 4-fold higher at 24, 36, and 48 h, while that emitted by pUC-TM8-G and pUC-TM9-G was similar to that of pUC-G, although slight differences were observed at 24 and 36 h. The *E. coli* cell pellets obtained after a 24 h-culture are shown in Fig 3. When these pellets were excited with a blue LED light source, fluorescence emission was observed from pUC-TM-G, pUC-TM5-G, and pUC-TM7-G, whereas that from pUC19, pUC-G, pUC-TM8-G, and pUC-TM9-G was relatively lower. The fluorescence of pUC-TM7-G was lower than that of pUC-TM-G and pUC-TM5-G. These results are consistent with the fluorescence intensity measurements, confirming that mUkG1 was expressed by pUC-TM-G, pUC-TM5-G, and pUC-TM7-G.

### Identification of the initiation codon for mUkG1

**Fig 4:**
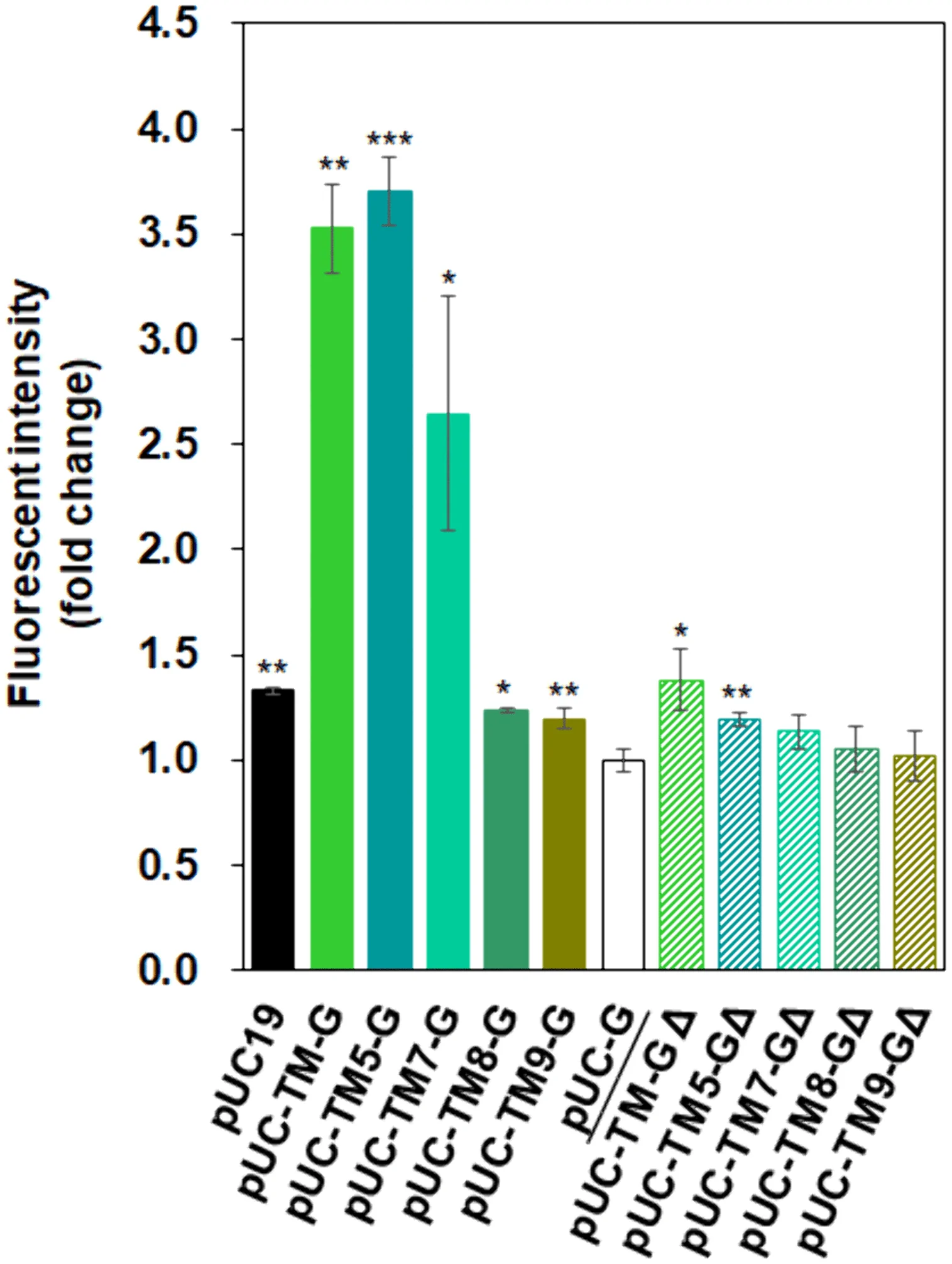
Comparison of fluorescence intensity exhibited by *E. coli* harboring pUC-TMx-G and pUC-TMx-GΔ. Fold changes in fluorescence intensities over the control, i.e., pUC-G (white bar with underlined label), were calculated after 24 h of incubation for each *E. coli* transformant. Data are presented as the mean ± standard deviation of three biological replicates. Significant differences from pUC-G are indicated by *P < 0.05, **P < 0.01, and ***P < 0.001.

**Fig 5:**
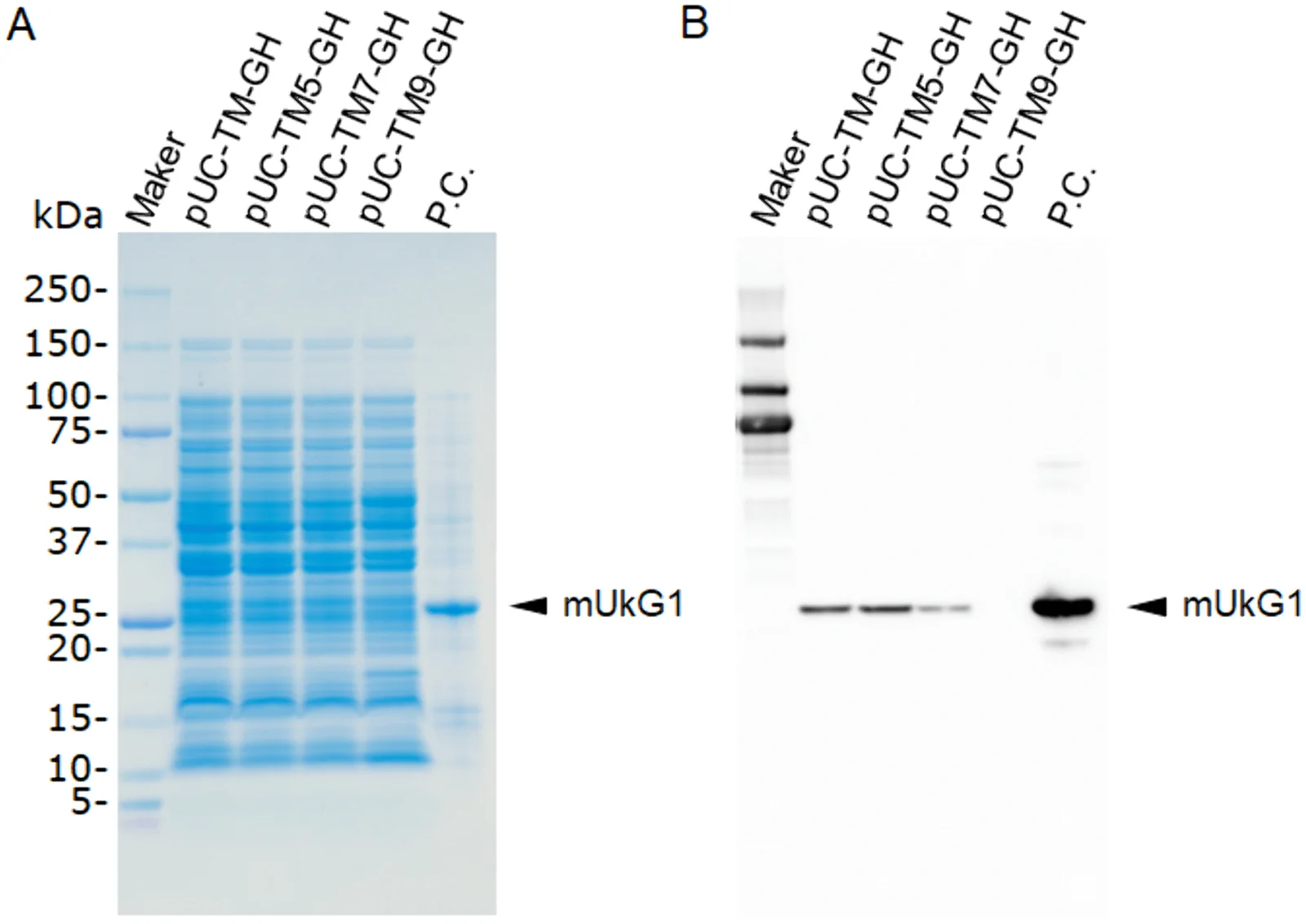
SDS-PAGE and Western blotting analyses of 6×His-tagged mUkG1 expressed in *E. coli* harboring pUC-TMx-GH after 24 h of culture. After measuring OD_600_, the cell pellet was collected from 1.5 mL of the culture in LB medium and lysed in 75 µL of BugBuster Master Mix. A 20 µL sample was prepared from each lysate, normalized to 8.5 divided by the OD_600_ value. Using 10 µl of each sample, (A) SDS-PAGE and (B) Western blotting analyses were performed using 2 µL of the lysate of the BL21 (DE3) *E. coli* strain expressing 6×His-tagged mUkG1 as the positive control. The 6×His-tagged mUkG1 is indicated by the solid arrowhead.

Despite the expression of mUkG1 in pUC-TM-G, pUC-TM5-G, and pUC-TM7-G, it was unknown whether mUkG1 proteins were translated from the correct initiation codon. Therefore, the initiation codon (ATG) of mUkG1 was replaced with AGT on pUC-TMx-G, and the fluorescence intensity of these mutants (denoted as pUC-TMx-GΔ) was measured after 24 h cultivation (Fig 4). Unlike pUC-TM-G, pUC-TM5-G and pUC-TM7-G, the fluorescence intensities for pUC-TM-GΔ, pUC-TM5-GΔ and pUC-TM7-GΔ were significantly weak, comparable to those for pUC19, pUC-G, pUC-TM8-G and pUC-TM9-G. Next, a 6×His-tag was fused to the C-terminal of mUkG1 in pUC-TM-G, pUC-TM5-G, pUC-TM7-G, and pUC-TM9-G (denoted as pUC-TMx-GH) for Western blotting analysis using an anti-6×His-tag antibody. After culturing for 24 h, the total protein from the transformants harboring pUC-TM-GH, pUC-TM5-GH, pUC-TM7-GH, and pUC-TM9-GH was analysed by SDS-PAGE and Western blotting. Bands corresponding to mUkG1 could not be identified for any of the transformants using Coomassie brilliant blue staining after SDS-PAGE, probably because of low expression of mUkG1 (Fig 5A). In contrast, Western blotting analysis clearly revealed 25.6-kDa bands corresponding to mUkG1 for pUC-TM-GH, pUC-TM5-GH, and pUC-TM7-GH, but not for pUC-TM9-GH (Fig 5B). These observations suggest that intact mUkG1 proteins with no fused peptide or nontruncation at their N-terminal were expressed; i.e., mUkG1 proteins were translated from the correct initiation codon.

### Promoter analysis

**Fig 6:**
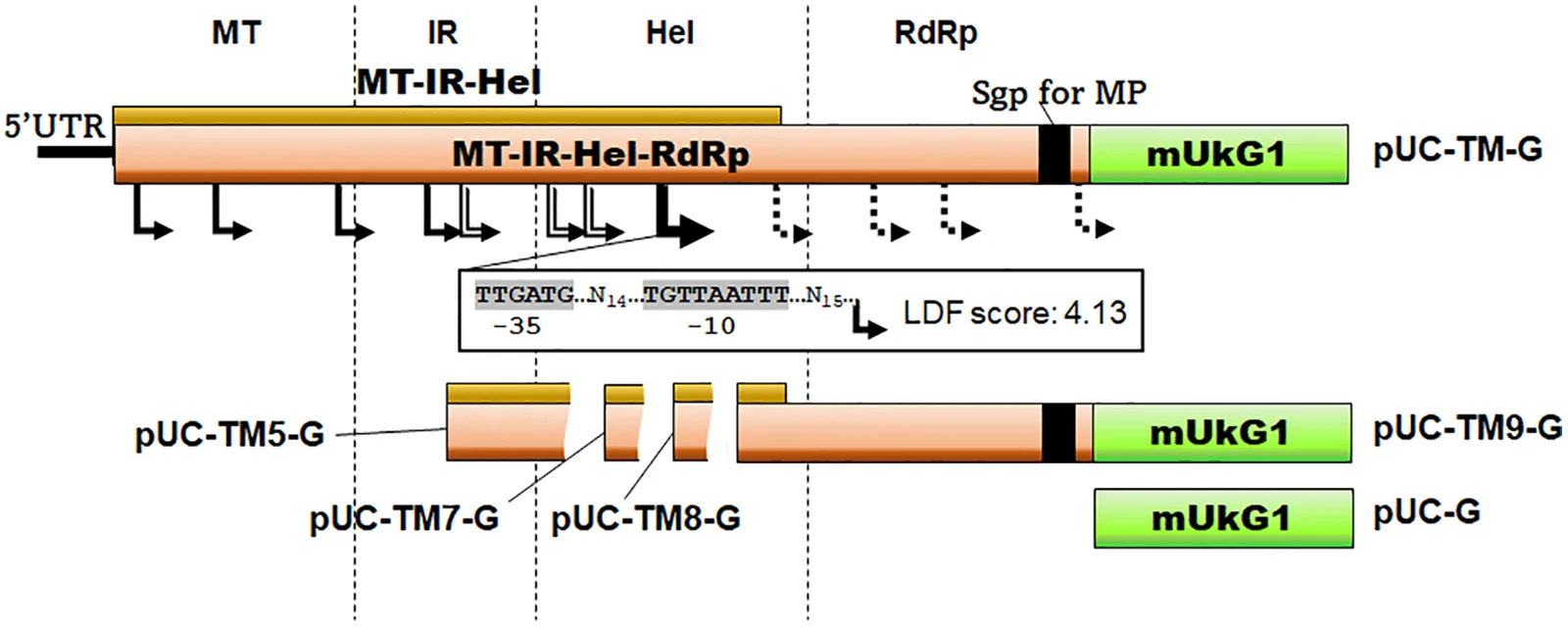
Prediction of sigma 70 promoters using the bacterial sigma-70 promoter recognition program (BPROM). The bent arrows indicate the transcription start sites of the predicted promoters. The solid and double lines indicate the regions within pUC-TM-G only and pUC-TM-G and pUC-TM5-G, respectively. The thick lines indicate the regions within pUC-TM-G, pUC-TM5-G, and pUC-TM7-G, while the dashed lines show the areas in all constructs. The core elements and LDF score of the candidate promoter are shown in the box.

All the plasmids constructed in this study lost the *lac*Z promoter derived from pUC19. Furthermore, because the modified TMV genes were cloned in a direction opposite to that of Amp^R^, the mUkG1 sense RNA was not transcribed even if the transcription readthrough for Amp^R^ expression occurs. This observation led to the hypothesis that the gene expression elements are located upstream of the mUkG1 gene in the modified TMV gene sequence, thereby leading to the transcription of mUkG1 mRNA. To confirm this hypothesis, a promoter prediction analysis was performed using the bacterial sigma-70 promoter recognition program (BPROM), yielding 80% accuracy and specificity [19]. Of the 12 putative promoter candidates (Table 1), one was located at the boundary region of TM7 and TM8 in the Hel domain, and therefore, was present only in pUC-TM-G, pUC-TM5-G, and pUC-TM7-G, which clearly expressed mUkG1 (Fig 6). The linear discriminant function (LDF) score was 4.13 (an LDF score ≥ 0.2 is considered significant) and the suggested core promoter elements were ‘TTGATG’ (−35 box) and ‘TGTTAATTT’ (-10 box). Four candidates with LDF scores of 4.37, 3.94, 2.86, and 2.68 were found in all modified TMV genomes, whereas three candidates with LDF scores of 2.69, 1.70, and 3.62 were detected only in pUC-TM-G and pUC-TM5-G. The remaining four candidates were found only in pUC-TM-G. To determine the 5’-end of the transcribed mRNA, 5’-RACE was also performed with mRNAs derived from pUC-TM5-G and pUC-TM8-G (S2 Fig). The 5’-ends of the PCR amplicons generated using the 5’-end-phosphorylated primer against the mUkG1 sequence, which were over 1 kb from the initiation codon of mUkG1, were barely detectable. This might be because the amplicon length exceeds the maximum elongation capacity of the reverse transcriptase. Therefore, 5’-RACE was performed again using a 5’-end-phosphorylated primer designed to anneal at sequences of 1877 nts upstream of the initiation codon for mUkG1. The 5’-ends located in the TM8 region (2810–3203 nts) were newly detected for pUC-TM5-G (S3 Fig). The most frequently detected 5’ end was at the 2828 nt position, which was located immediately downstream of the predicted promoter at the boundary region of TM7 and TM8.

**Table 1:** Promoter prediction using the bacterial sigma-70 promoter recognition program.

Location	TSS position	LDF score	−35 box		−10 box		TF binding site		
			Sequence	Position	Sequence	Position	Sequence	Position	TF
TM	78	2.16	TTACAA	45	AATTACAAT	62			
	618	1.35	TTCCTG	586	TGTCACAAT	603			
	1013	3.57	TTAATA	976	TTCTAGGAT	998			
	1423	2.50	TGGGAT	1389	TGTTACAAT	1408			
TM TM5	1749	2.69	TGGAAG	1717	ATGTACAAT	1734			
	2080	1.70	TTGCTG	2041	TCCTATTCT	2064			
	2397	3.62	TTGAAA	2365	AAGTATCAT	2382			
TM TM5 TM7	2828	4.13	TTGATG	2793	TGTTAATTT	2813	TTAATTTT	2815	rpoD18
							AATTTTCT	2817	rpoD17
TM TM5 TM7 TM8 TM9	3359	2.86	TTGTTA	3325	TAGTATCAT	3344			
	3858	4.37	TTGCTT	3819	AATAAAAAT	3843	TTTTTTGA	3804	fnr
							AAATAAAA	3842	ihf
							AATAAAAA	3843	arcA
							ATAAAAAT	3844	argR
	4282	2.68	ATCTCA	4244	CAGTAGAAT	4267			
	4898	3.94	TTGTCT	4866	TTTTAGAAG	4883	TTATAAAA	4844	rpoD16
							TTGTCTGA	4866	rpoD17
							GTCTGATA	4868	rpoE
							TTCTTTTT	4879	ompR

TSS, LDF, and TF represent transcription start site, linear discriminant function, and transcription factor, respectively.

**Fig 7:**
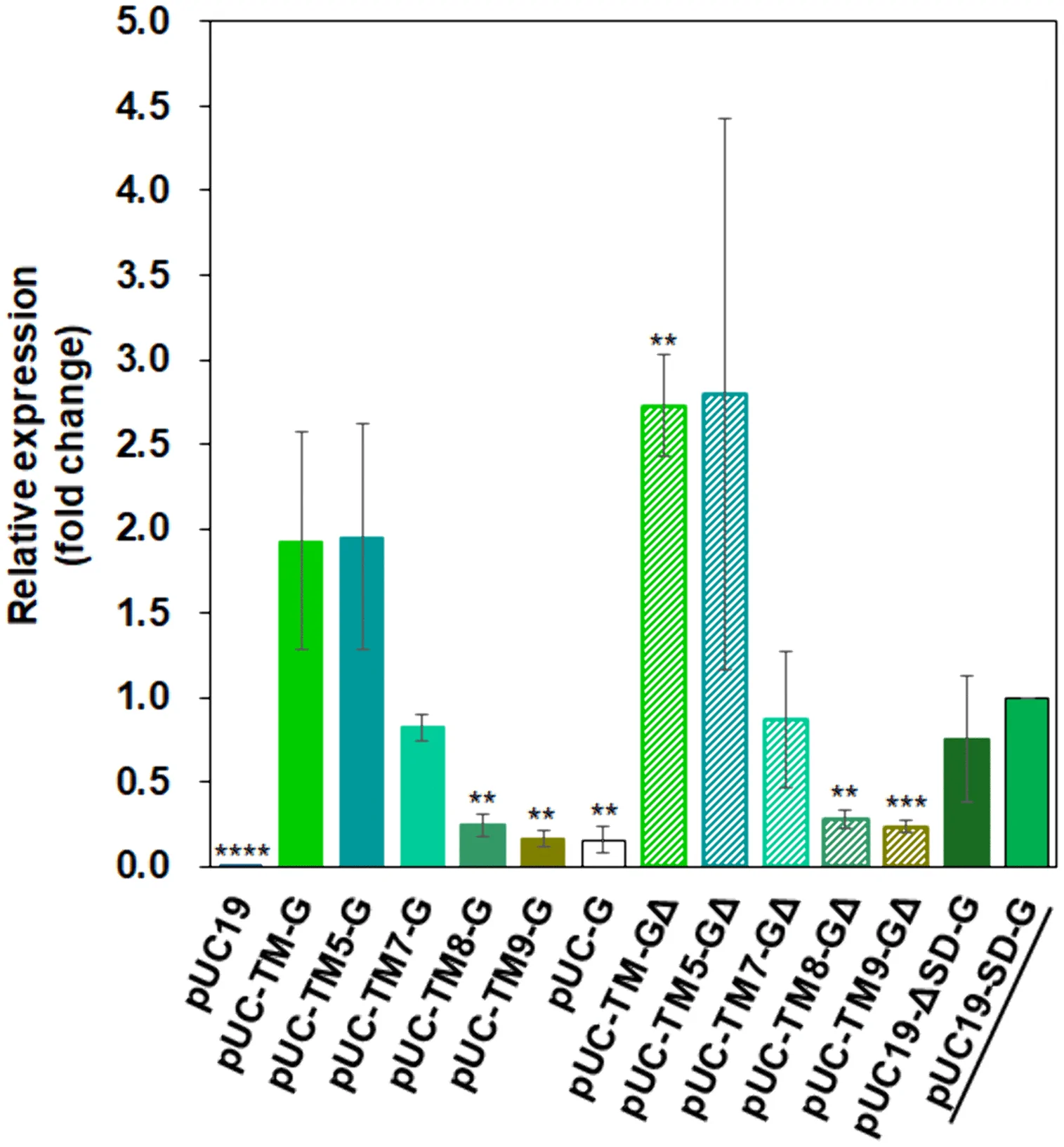
RT-qPCR analysis of the mRNAs derived from *E. coli* harboring pUC-TMx-G, pUC-TMx-GΔ, pUC19-SD-G and pUC19-ΔSD-G. The relative expression levels of mRNAs for mUkG1 over the control, i.e., pUC19-SD-G (green bar with underlined label), were calculated using the ΔΔCt method. Data are presented as the mean ± standard deviation of three biological replicates. Significant differences from pUC-G are indicated by **P < 0.01, ***P < 0.001 and ****P < 0.0001.

**Fig 8:**
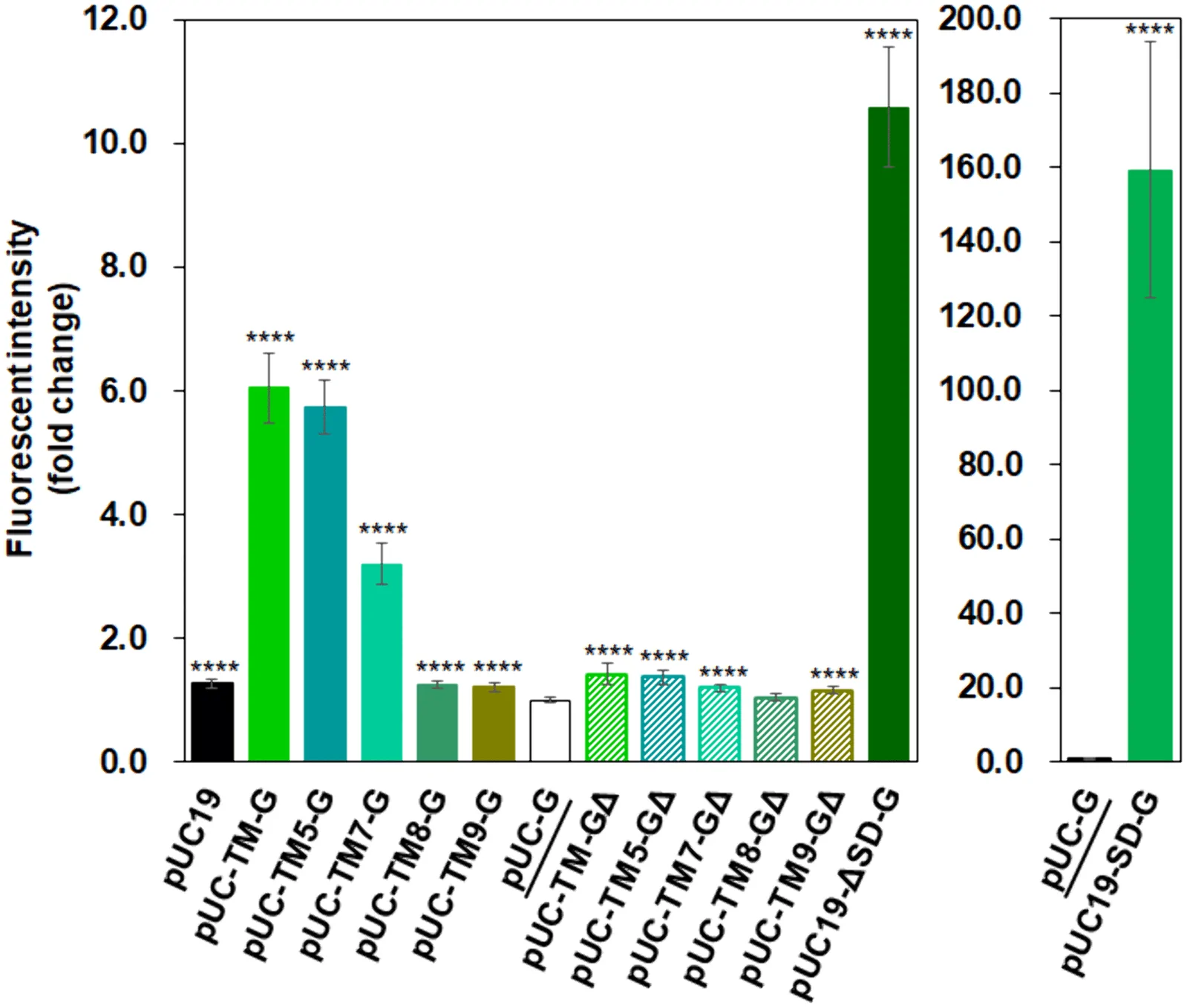
Comparison of fluorescence intensity exhibited by *E. coli* harboring pUC-TMx-G, pUC-TMx-GΔ, pUC19-SD-G and pUC19-ΔSD-G. Fold changes in fluorescence intensities over the control, i.e., pUC-G (white bar with underlined label), were calculated after 24 h of incubation for each *E. coli* transformant. Data are presented as the mean ± standard deviation of three biological replicates. Significant differences from pUC-G are indicated by ****P < 0.0001.

Next, the transcriptional activity of the promoter identified in the cDNA was compared with that of the *lac* promoter from pUC19. Two reference vectors, pUC19-SD-G and pUC19-ΔSD-G, were constructed by replacing the sequence from the initiation codon to the *Xba*I site of *lacZα* in pUC19 with the mUkG1-encoding sequence (S4 Fig). In pUC19-ΔSD-G, the SD sequence (AGGAA) was replaced with its complementary sequence (TCCTT). RT-qPCR analysis confirmed promoter activity in pUC-TM-G, pUC-TM5-G, and pUC-TM7-G, and the transcriptional activity was not significantly different from that of the *lac* promoter (Fig 7). When the fluorescence intensities of *E. coli* HST08 cells harboring the two reference vectors were compared with those of cells harboring pUC-TMx-G, pUC19-SD-G showed a 24-fold higher fluorescence intensity, whereas pUC19-ΔSD-G showed a 1.6-fold higher fluorescence intensity than pUC-TM-G and pUC-TM5-G (Fig 8). These observations indicate that the reduced translational output is attributable not to promoter activity but rather to the absence of the SD sequence and the extremely long 5’-UTR.

### Transcription of modified cDNAs of the TMV genome by inducible promoters

**Fig 9:**
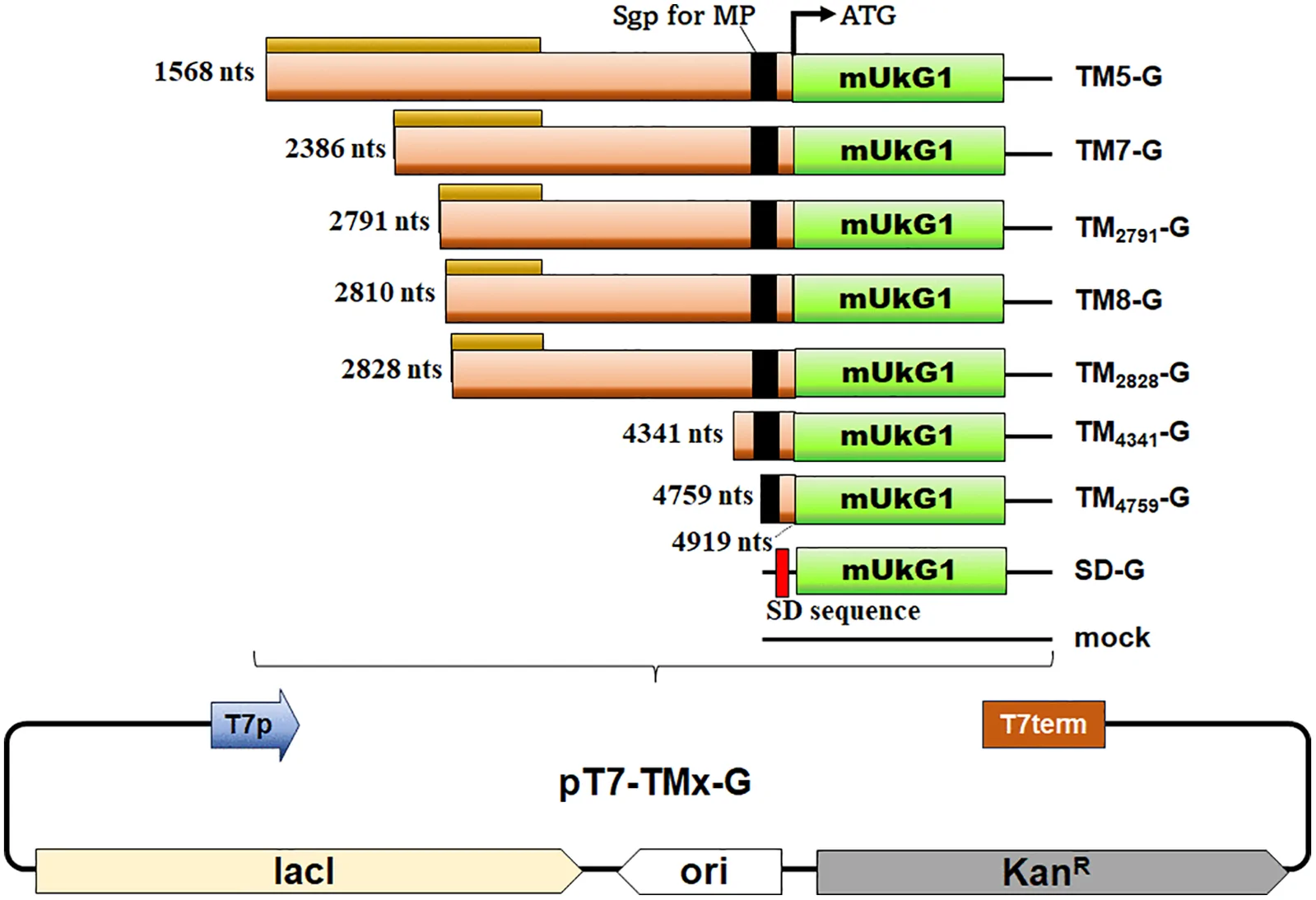
Construction of the pT7-TMx-G vectors. The fragments of the 5’-end-truncated mutants and SD-attached mUkG1 were cloned just downstream of the T7 promoter in the pET26b backbone.

**Fig 10:**
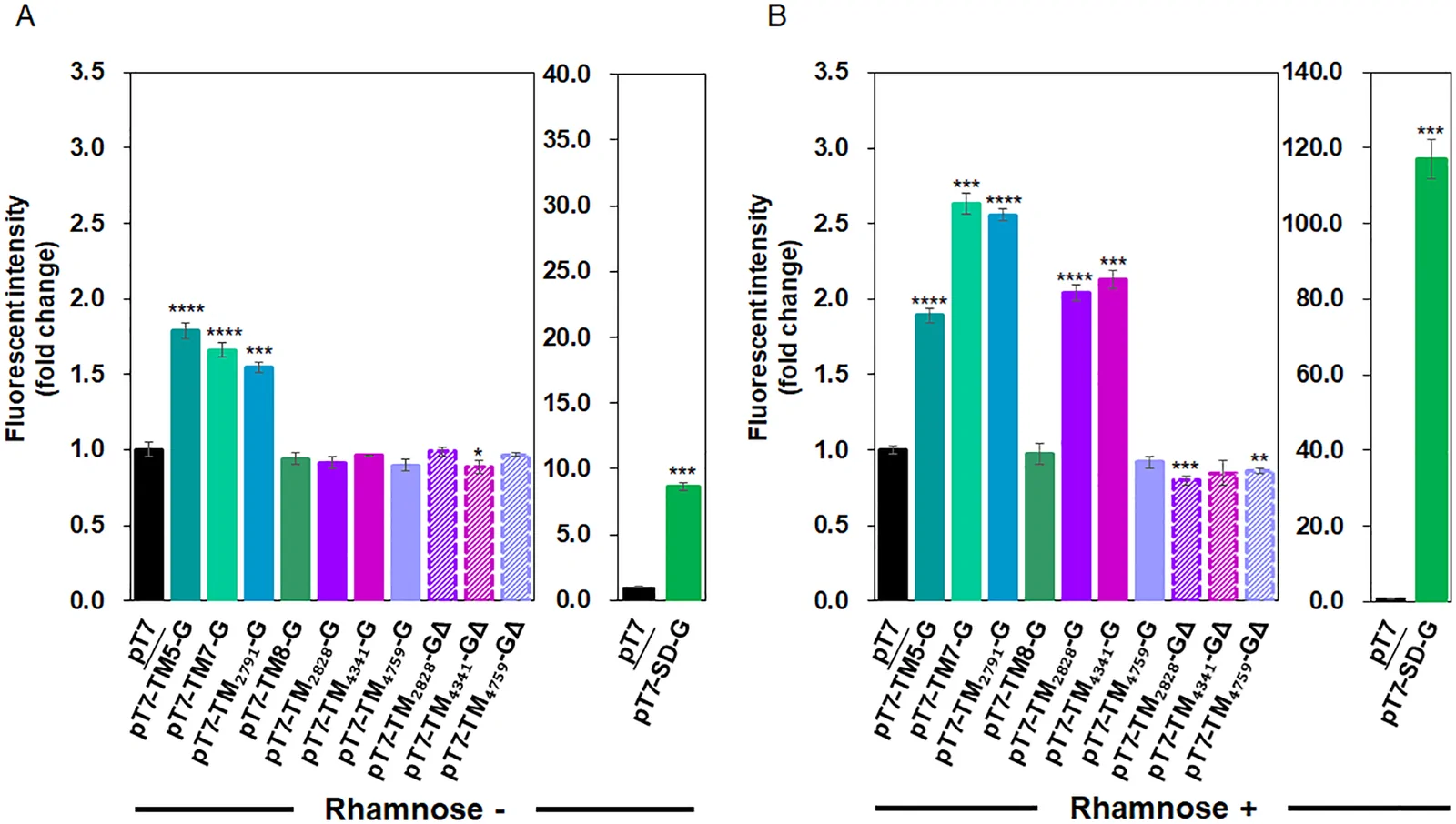
Fluorescence intensity analysis of *E. coli* harboring the pT7-TMx-G vectors under uninduced and induced conditions. Fold changes in fluorescence intensities over the control, pT7 (black bar with underlined label), were calculated for each *E. coli* transformant after 10 h of culture under (A) uninduced and (B) induced (6 h after rhamnose induction following a 4-h culture period) conditions. Data are presented as the mean ± standard deviation of three biological replicates. Significant differences from the control are indicated by *P < 0.05, **P < 0.01, ***P < 0.001, and ****P < 0.0001.

To confirm mUkG1 translation from long mRNAs, which are transcribed from 2096 nts upstream of the initiation codon for mUkG1, bacteriophage T7 promoter-inducible vectors were constructed (Fig 9). All the expression vectors, pT7-TM5-G, pT7-TM7-G, and pT7-TM_2791_-G, contain the predicted promoter sequence in the Hel domain, while the pT7-TM_2828_-G, pT7-TM8-G, pT7-TM_4341_-G and pT7-TM_4759_-G vectors do not. Based on the 5’-RACE results, pT7-TM_2828_-G, pT7-TM_4341_-G, and pT7-TM_4759_-G were constructed to generate mRNAs transcribed from the detected 5’-ends. pT7-SD-G and pT7 were used as positive and negative controls, respectively. Constructs harboring mutated initiation codons included pT7-TM_2828-_GΔ, pT7-TM_4341_-GΔ, and pT7-TM_4759_-GΔ. These vectors were transformed into the *E. coli* KRX strain, which was cultivated in LB medium. Fluorescence intensity was measured under both uninduced (after 10 h of culture) and induced (6 h after rhamnose induction following a 4-h culture period) conditions. Under the no-induction condition, significant fluorescence intensity was detected from pT7-TM5-G, pT7-TM7-G, pT7-TM_2791_-G, and pT7-SD-G. pT7-TM8-G, pT7-TM_2828_-G, pT7-TM_4341_-G, pT7-TM_4759_-G, pT7-TM_2828-_GΔ, pT7-TM_4341_-GΔ, pT7-TM_4759_-GΔ did not exhibit fluorescence (Fig 10A). Probably, the fluorescens for pT7-SD-G is from basal expression of mUkG1. As expected, pT7-TM_2791_-G, which contains the predicted promoter, exhibited fluorescence emission despite a difference of only 20 nts between pT7-TM_2791_-G and pT7-TM8-G. Under the induced condition, fluorescence intensity increased for pT7-TM_2828_-G and pT7-TM_4341_-G in addition to pT7-TM5-G, pT7-TM7-G, pT7-TM_2791_-G, and pT7-SD-G. In contrast, no increase in fluorescence intensity was observed for pT7-TMx-GΔs. These results indicate that the inducible promoter enhances mRNA transcription, resulting in an increased expression of mUkG1 along with the translation of mUkG1 from the correct initiation codon, irrespective of the length of 5’-UTR (Fig 10B). The vectors, pT7-TM8-G and pT7-TM_4759_-G, did not display fluorescence even after induction. It might be because pT7-TM8-G and TM_4759_-G have a TT sequence at positions +2 and +3 of the T7 promoter, which showed significantly lower activity in the T7 promoter variants randomized from positions +2 to +16 [20]. In a preliminary examination, pT7-TM_2809_-G and pT7-TM_4758_-G, which harbor the GT sequence at positions +2 and +3, showed increased fluorescence intensity upon induction (S5 Fig).

## Discussion

In this study, cDNA fragments were generated from the TMV RNA genome by replacing the MP gene and its downstream region with a gene encoding the green fluorescent protein mUkG1. Intact mUkG1 proteins were expressed from pUC-TM-G and pUC-TM7-G without additional or missing amino acids. Moreover, the Hel domain-coding region contributed to mUkG1 expression in *E. coli* and was predicted to contain a sigma 70 promoter with an LDF score of 4.13. Because the mRNA derived from this promoter contributes to mUkG1 expression, the 5’-UTR is expected to span 2096 nts, which is extremely long. In *E. coli*, the 5’-UTR is usually 25–35 nts long [21]. Expression analyses using pUC-TMx-G and pT7-TMx-G demonstrated that the predicted promoter functions appropriately and that intact mUkG1 is expressed irrespective of the length of the 5’-UTR of the mUkG1 mRNA.

SD-independent translation in *E. coli* has been reported in the RNA genomes of several plant RNA viruses, such as TMV RNA and Alfalfa Mosaic Virus RNA 4 [16,17]. The Ω- and U-rich sequences in the 5’-UTRs are recognized by the ribosomal protein S1 of *E. coli*, resulting in SD-independent translation [17]. S1 has high affinity for U-rich or A/U-rich sequences [22,23] and supports the binding of the 30S subunit to mRNAs without requiring an SD sequence [24]. Bioinformatics analyses have shown that the sequences surrounding the initiation codons of mRNAs without an SD sequence form significantly weaker secondary structures than those with an SD sequence [25,26]. In the modified TMV cDNA, the 80 nts (containing RdRp-encoding gene) upstream of the initiation codon of mUkG1 had an AT content of 70%, which was significantly higher than the *E. coli* genome, which has approximately 49% AT content [27–29]. In addition, analysis of SD sequence 4-mers (AGGA|GGAG|GAGG), 5-mers (AGGAG|GGAGG), or 6-mers (AGGAGG) indicated that there was only one SD sequence, GGAGG, at 111 nts upstream of the initiation codon. This GGAGG sequence was located in the Sgp region of MP in the RdRp-encoding gene, where the negative strand forms two stem-loop structures [10]. This led to the prediction of a stem-loop structure involving GGAGG in the positive strand. Therefore, the lower expression of intact mUkG1 may have been driven in an SD-independent manner. Additionally, the stem-loop structures and adjacent A/U-rich region may function as a ribosomal standby site [30], as the rhamnose-induced increase in fluorescence intensity was significantly lower for pT7-TM_4758_-G (S5 Fig), in which 15 nts at the 5’-end of the MP Sgp region were deleted, potentially destabilizing the stem-loop structures.

Some eukaryotic promoters, such as the B33 promoter and the ST-LSI promoter of *Solanum tuberosum*, can be recognized by *E. coli* [31,32]. Moreover, random DNA sequences from *S. cerevisiae* can initiate gene expression in *E. coli* [33]. Studies have shown that the promoters of eukaryotic viruses, such as Cauliflower mosaic virus, Simian vacuolating virus 40, and human immunodeficiency virus type 1, can drive gene expression in *E. coli* [32,34]. These viruses are either DNA or RNA viruses with a reverse transcriptase (RT) that requires the transcription of DNA into RNA to replicate in host cells. In turn, TMV is an RNA virus that lacks RT and does not require a DNA intermediate for replication. Therefore, it was surprising that the promoter region recognized by *E. coli* in the cDNA of the TMV RNA genome is functional. These results indicate that the genomes of RNA viruses potentially hide gene expression elements that function in prokaryotic cells.

The crystal structure of ToMV-Hel, revealed using X-ray crystallography, was shown to consist of an N-terminal domain and two RecA-like domains (denoted as 1A and 2A), which form a helicase core [35]. As the primary amino acid sequences of TMV-Hel and ToMV-Hel demonstrate 89.6% homology, a structural model of TMV-Hel has been constructed based on the ToMY-Hel structure [36]. In this model, the predicted promoter is located just downstream of the putative Walker B motif (RLFIDE at nts 2772–2789) of the RecA-like 1A domain: 261 nts upstream of the encoding gene of the RecA-like 2A domain, immediately followed by the RdRp domain. RecA mediates homologous recombination and genetic repair in almost all bacteria. [37,38]. RecA and RecA-like proteins have been found in several prokaryotes and eukaryotes and are believed to have evolved from a single common ancestor of cellular organisms [39].

Among RNA viruses, the RdRp-encoding genes are the only universally conserved genes. The viral RdRps belong to polymerases containing palm catalytic domains [40,41]. The phylogenetic tree constructed for RdRp based on multiple-sequence alignments suggested that the last common ancestors of +RNA viruses encoded only two proteins, the RdRp and a single jelly-roll CP, and that additional genes (such as helicase- and capping enzyme-encoding genes) were independently captured in subsequent evolution steps [4]. Furthermore, the RdRps of +RNA viruses and the RTs of group II introns, which are widespread in bacterial genomes, were shown to be highly similar [1,4,42]. In the TMV genome, a termination codon was inserted between the MT-Hel and the RdRp domains, implying that the functions of MT and Hel were acquired separately from the RdRp in different evolutionary steps. The extremely long 5’-UTR may arise from a coincidental combination of the promoter trace with an A/T-rich sequence in the 3’-terminal region of the RdRp-coding gene. Taken together, the prokaryotic characteristics in the TMV genome are as follows: (i) the promoter sequence was hidden upstream of Rec-like 2A domain of TMV-Hel, (ii) a local RNA unfolding mechanism, mediated by A/T-rich sequences, potentially contributed to the recognition of the correct initiation codon of mUkG1 (as substitute for MP) without an SD sequence in *E. coli*, and (iii) the RdRps of RNA viruses might share a common ancestor with RTs in group II introns. These features suggest that ancestral prokaryotic gene elements significantly contribute to the origin and evolution of highly diverse and abundant RNA viruses found in the virome of eukaryotes. Recent metagenomic studies suggest a much greater diversity of RNA viruses in prokaryotes than that described previously [43,44]. In addition, an evolutionary relationship between arthropod and plant viruses belonging to the families *Virgaviridae* and *Kitaviridae* has been proposed [45]. Bacterial endosymbionts are widespread in insects, particularly in sap-feeding species, many of which serve as vectors of plant diseases [46–48]. Thus, the presence of an *E. coli*-recognizable promoter sequence in the TMV genome may reflect evolutionary processes involving horizontal gene transfer among arthropods, their microbial partners, and viruses. Analysis of the genome structure of newly discovered prokaryotic RNA viruses [49,50] and hidden gene elements in RNA genomes using cDNAs would facilitate a deeper understanding of the origin and evolution of RNA viruses.

## Supporting information

S1 Fig Schematic representation of the vector construction strategy. (A) A fragment of the mUkG1-encoding gene was digested using *Asc*I and then cloned into the *Zra*I and *Asc*I sites of pCLY-TM. (B) Next, the PCR products of the modified TMV gene, its truncated mutants, and mUkG1 were digested using *Avr*II and cloned into the *Sma*I and *Xba*I sites of pUC19. (C) Finally, the fragments of the modified TMV gene, its truncated mutants, and mUkG1 were amplified by PCR using primers with 15-base extensions complementary to the vector. The PCR fragments were cloned into pUC19 between both *Pvu*II sites using the In-Fusion cloning kit.
(TIF)

S2 Fig 5’-RACE analysis for pUC-TM5-G and pUC-TM8-G using a primer specific to the mUkG1 sequence. The 5’-ends of the cDNA sequences from 73 and 61 clones for pUC-TM5-G and pUC-TM8-G, respectively, were determined. The bent and blue arrows indicate the nucleotide and annealing positions of the 5’-end-phosphorylated primer, respectively.
(TIF)

S3 Fig 5’-RACE analysis for pUC-TM5-G and pUC-TM8-G using a primer specific to 1877 nts upstream of the initiation codon of mUkG1. The 5’-ends of the cDNAs from 43 and 31 clones for pUC-TM5-G and pUC-TM8-G, respectively, were determined. The bent and blue arrows show the nucleotide and annealing positions of the 5’-end-phosphorylated primer, respectively.
(TIF)

S4 Fig Construction of the pUC19-SD-G and pUC19-ΔSD-G vectors. The N-terminal region of the LacZα peptide in the pUC19 was replaced with mUkG1. In pUC19-ΔSD-G, the SD sequence was replaced with its complementary sequence.
(TIF)

S5 Fig Fluorescence intensity analysis of *E. coli* harboring pT7-TM_2809_-G, TM8-G, TM_4758_-G, TM_4759_-G, and SD-G vectors under uninduced and induced conditions. Fold changes in fluorescence intensities over the control, pT7 (black bar with underlined label), were calculated for each *E. coli* transformant after 10 h of culture under (A) uninduced and (B) induced (6 h after rhamnose induction following a 4-h culture period) conditions. Data are presented as the mean ± standard deviation of three biological replicates. Significant differences from the control are indicated by *P < 0.05, **P < 0.01, and ***P < 0.001.
(TIF)

S1 Table Sequences of TMV fragments and mUkG1. Homologous sequences between two fragments connected by homologous recombination and restriction enzyme sites are indicated by the underlined texts and grey boxes, respectively.
(DOCX)

S2 Table Primer list. The primers used to construct different vectors and for 5’-RACE and RT-qPCR analysis are listed.
(DOCX)

S3 Table Promoter sequences. The T7 promoter was connected immediately upstream of each cDNA without any nucleotide insertions.
(DOCX)

S1 Raw images Original gel image and blot for Fig 5. (PDF)

S1 Dataset All plotted datapoints, organized by figure in Excel file. (XLSX)
