# Supplementary material for: Detection of bacterial gene expression elements on Tobacco mosaic virus RNA using cDNA analysis
Source: PLoS One. 2026 Sep 15;21(9):e0358128. doi: 10.1371/journal.pone.0358128 (PMC13577412; doi:10.1371/journal.pone.0358128)
Supplement: Supplementary file 1 [file pone.0358128.s009.pdf]

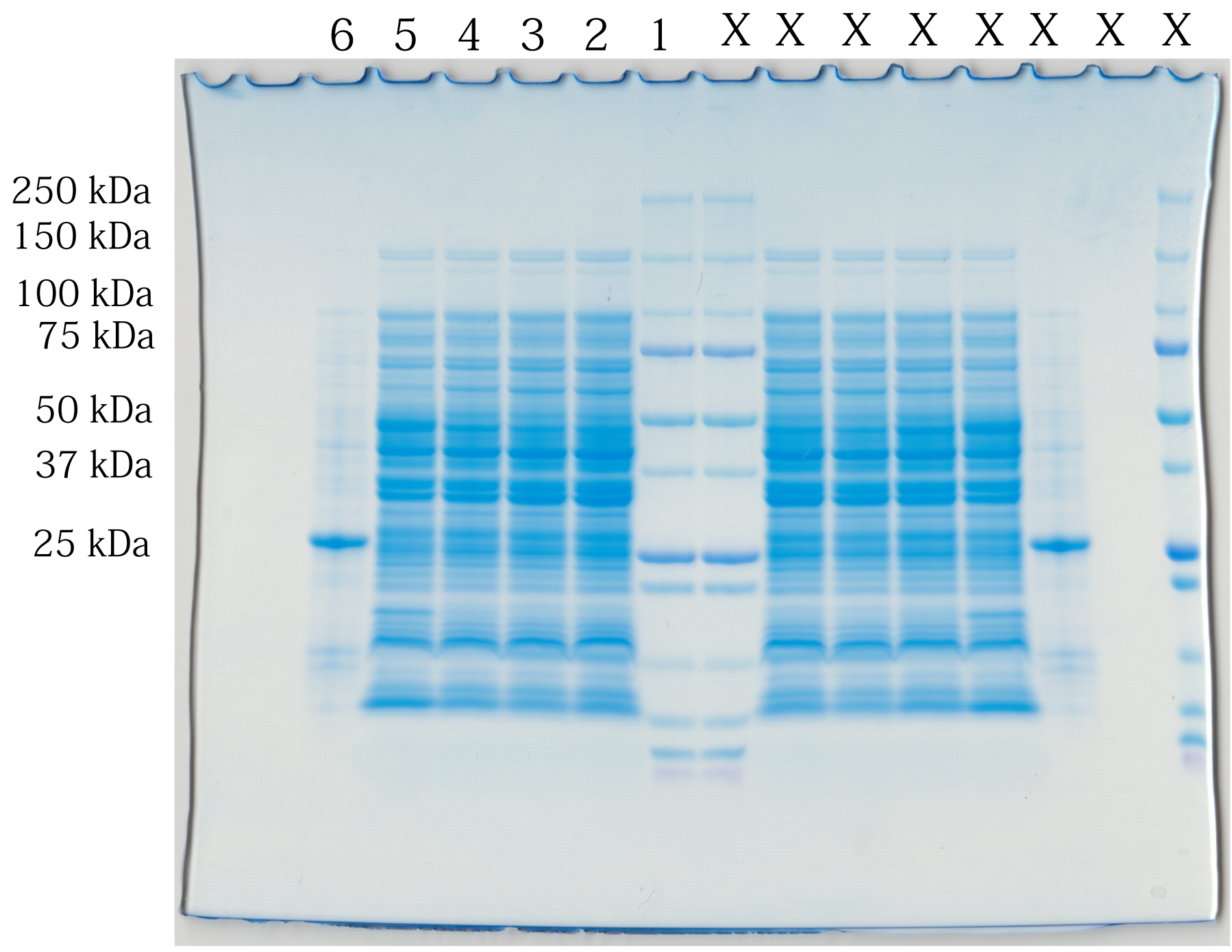

Loading: total-cell lysate
1: Precision Plus Protein Dual Xtra Prestained Protein Standards
2: *E. coli* harboring pUC-TM-GH
3: *E. coli* harboring pUC-TM5-GH
4: *E. coli* harboring pUC-TM7-GH
5: *E. coli* harboring pUC-TM9-GH
6: *E. coli* harboring pET26b-mUkG1

The total proteins were visualized by CBB staining and then imaged as tiff by graphics scanner from EPSON.

This image was used to create figure panel 5A.

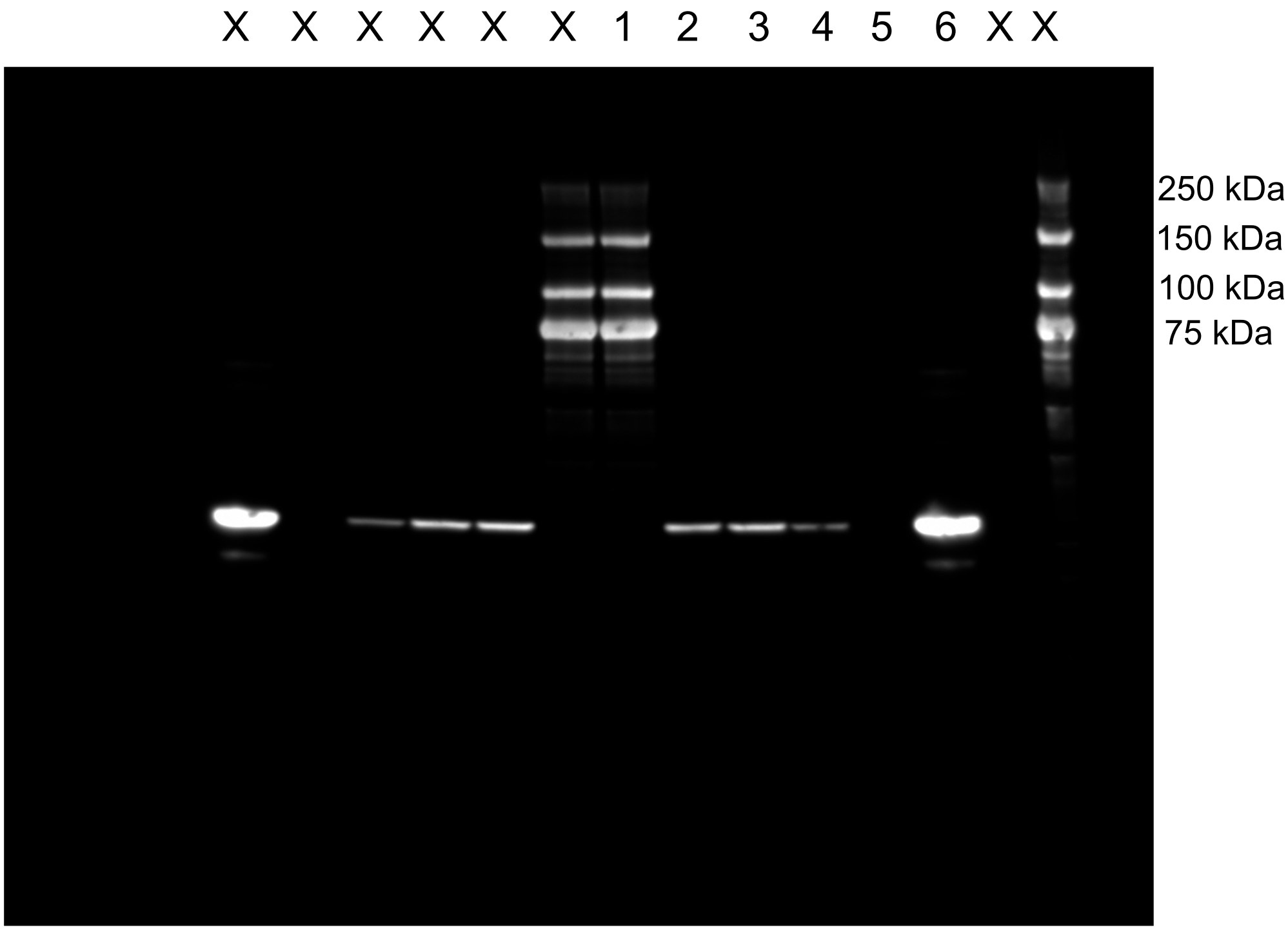

Loading: total-cell lysate
1: Precision Plus Protein Dual Xtra Prestained Protein Standards
2: *E. coli* harboring pUC-TM-GH
3: *E. coli* harboring pUC-TM5-GH
4: *E. coli* harboring pUC-TM7-GH
5: *E. coli* harboring pUC-TM9-GH
6: *E. coli* harboring pET26b-mUkG1

The 6xHis-tagged mUkG1 was visualized by Clarity Western ECL Substrate and then imaged as tif by LuminoGraph from ATTO.

This image was used to create figure panel 5B after inverting in Photoshop Elements.
