# Supplementary material for: Detection of bacterial gene expression elements on Tobacco mosaic virus RNA using cDNA analysis
Source: PLoS One. 2026 Sep 15;21(9):e0358128. doi: 10.1371/journal.pone.0358128 (PMC13577412; doi:10.1371/journal.pone.0358128)
Supplement: Supplementary file 2 [file pone.0358128.s006.docx]

**S1 Table**

**TM1 (454 bp)**

GGAACAAAAGCTGGCTAGAACTAGTGGATCCCAAGCTTGGCCGAGCTCAGCGGCCGCTTGGAATTCTACGTATTTTTACAACAATTACCAACAACAACAAACAACAAACAACATTACAATTACTATTTACAATTACAATGGCATACACACAGACAGCTACCACATCAGCTTTGCTGGACACTGTCCGAGGAAACAACTCCTTGGTCAATGATCTAGCAAAGCGTCGTCTTTACGACACAGCGGTTGAAGAGTTTAACGCTCGTGACCGCAGGCCCAAGGTGAACTTTTCAAAAGTAATAAGCGAGGAGCAGACGCTTATTGCTACCCGTGCGTATCCAGAGTTCCAAATTACATTTTATAACACGCAAAATGCCGTGCATTCGCTTGCAGGTGGATTGCGATCTTTAGAACTGGAATATCTGATGATGCAAATTCCCTACGGATCATTGACTTA

**TM2 (454 bp)**

GATGCAAATTCCCTACGGATCATTGACTTATGACATAGGCGGGAATTTTGCATCGCATCTGTTCAAGGGACGAGCATATGTACACTGCTGCATGCCCAACCTGGACGTTCGAGACATCATGCGGCACGAAGGCCAGAAAGACAGTATTGAACTATACCTTTCTAGGCTAGAGAGAGGGGGGAAAACAGTCCCCAACTTCCAAAAGGAAGCATTTGACAGATACGCAGAAATTCCTGAAGACGCTGTCTGTCACAATACTTTCCAGACAATGCGACATCAGCCGATGCAGCAATCAGGCAGAGTGTATGCCATTGCGCTACACAGCATATATGACATACCAGCCGATGAGTTCGGGGCGGCACTCTTGAGGAAAAATGTCCATACGTGCTATGCCGCTTTCCACTTCTCTGAGAACCTGCTTCTTGAAGATTCATACGTCAATTTGGACGAAATC

**TM3 (454 bp)**

GCTTTCCACTTCTCTGAGAACCTGCTTCTTGAAGATTCATACGTCAATTTGGACGAAATCAACGCGTGTTTTTCGCGCGATGGAGACAAGTTGACCTTTTCTTTTGCATCAGAGAGTACTCTTAATTATTGTCATAGTTATTCTAATATTCTTAAGTATGTGTGCAAAACTTACTTCCCGGCCTCTAATAGAGAGGTTTACATGAAGGAGTTTTTAGTCACCAGAGTTAATACCTGGTTTTGTAAGTTTTCTAGGATAGATACTTTTCTTTTGTACAAAGGTGTGGCCCATAAAAGTGTAGATAGTGAGCAGTTTTATACTGCAATGGAAGACGCATGGCATTACAAAAAGACTCTTGCAATGTGCAACAGCGAGAGAATCCTCCTTGAGGATTCATCATCAGTCAATTACTGGTTTCCCAAAATGAGGGATATGGTCATCGTACCATTATTCG

**TM4 (454 bp)**

TGAGGGATATGGTCATCGTACCATTATTCGACATTTCTTTGGAGACTAGTAAGAGGACGCGCAAGGAAGTCTTAGTGTCCAAGGATTTCGTGTTTACAGTGCTTAACCACATTCGAACATACCAGGCGAAAGCTCTTACATACGCAAATGTTTTGTCCTTTGTCGAATCGATTCGATCGAGGGTAATCATTAACGGTGTGACAGCGAGGTCCGAATGGGATGTGGACAAATCTTTGTTACAATCCTTGTCCATGACGTTTTACCTGCATACTAAGCTTGCCGTTCTAAAGGATGACTTACTGATTAGCAAGTTTAGTCTCGGTTCGAAAACGGTGTGCCAGCATGTGTGGGATGAGATTTCGCTGGCGTTTGGGAACGCATTTCCCTCCGTGAAAGAGAGGCTCTTGAACAGGAAACTTATCAGAGTGGCAGGCGACGCATTAGAGATCAGGGT

**TM5 (454 bp)**

AGAGAGGCTCTTGAACAGGAAACTTATCAGAGTGGCAGGCGACGCATTAGAGATCAGGGTGCCTGATCTATATGTGACCTTCCACGACAGATTAGTGACTGAGTACAAGGCCTCTGTGGACATGCCTGCGCTTGACATTAGGAAGAAGATGGAAGAAACGGAAGTGATGTACAATGCACTTTCAGAGTTATCGGTGTTAAGGGAGTCTGACAAATTCGATGTTGATGTTTTTTCCCAGATGTGCCAATCTTTGGAAGTTGACCCAATGACGGCAGCGAAGGTTATAGTCGCGGTCATGAGCAATGAGAGCGGTCTGACTCTCACATTTGAACGACCTACTGAGGCGAATGTTGCGCTTGCTTTACAGGATCAAGAGAAGGCTTCAGAAGGTGCTTTGGTAGTTACCTCAAGAGAAGTTGAAGAACCGTCCATGAAGGGTTCGATGGCCAGAGGA

**TM6 (454 bp)**

CCGTCCATGAAGGGTTCGATGGCCAGAGGAGAGTTACAATTAGCTGGTCTTGCTGGAGATCATCCGGAGTCGTCCTATTCTAAGAACGAGGAGATAGAGTCTTTAGAGCAGTTTCATATGGCAACGGCAGATTCGTTAATTCGTAAGCAGATGAGCTCGATTGTGTACACGGGTCCGATTAAAGTTCAGCAAATGAAAAACTTTATCGATAGCCTGGTAGCATCACTATCTGCTGCGGTGTCGAATCTCGTCAAGATCCTCAAAGATACAGCTGCTATTGACCTTGAAACCCGTCAAAAGTTTGGAGTCTTGGATGTTGCATCTAGGAAGTGGTTAATCAAACCAACGGCCAAGAGTCATGCATGGGGTGTTGTTGAAACCCACGCGAGGAAGTATCATGTGGCGCTTTTGGAATATGATGAGCAGGGTGTGGTGACATGCGATGATTGGAGAA

**TM7 (454 bp)**

ATCATGTGGCGCTTTTGGAATATGATGAGCAGGGTGTGGTGACATGCGATGATTGGAGAAGAGTAGCTGTCAGCTCTGAGTCTGTTGTTTATTCCGACATGGCGAAACTCAGAACTCTGCGCAGACTGCTTCGAAACGGAGAACCGCATGTCAGTAGCGCAAAGGTTGTTCTTGTGGACGGAGTTCCGGGCTGTGGGAAAACCAAAGAAATTCTTTCCAGGGTTAATTTTGATGAAGATCTAATTTTAGTACCTGGGAAGCAAGCCGCCGAAATGATCAGAAGACGTGCCAATTCCTCAGGGATTATTGTGGCCACGAAGGACAACGTTAAAACCGTTGATTCTTTCATGATGAATTTTGGGAAAAGCACACGCTGTCAGTTCAAGAGGTTATTCATTGATGAAGGGTTGATGTTGCATACTGGTTGTGTTAATTTTCTTGTGGCGATGTCATT

**TM8 (454 bp)**

TTGTGTTAATTTTCTTGTGGCGATGTCATTGTGCGAAATTGCATATGTTTACGGAGACACACAGCAGATTCCATACATCAATAGAGTTTCAGGATTCCCGTACCCCGCCCATTTTGCCAAATTGGAAGTTGACGAGGTGGAGACACGCAGAACTACTCTCCGTTGTCCAGCCGATGTCACACATTATCTGAACAGGAGATATGAGGGCTTTGTCATGAGCACTTCTTCGGTTAAAAAGTCTGTTTCGCAGGAGATGGTCGGCGGAGCCGCCGTGATCAATCCGATCTCAAAACCCTTGCATGGCAAGATCCTGACTTTTACCCAATCGGATAAAGAAGCTCTGCTTTCAAGAGGGTATTCAGATGTTCACACTGTGCATGAAGTGCAAGGCGAGACATACTCTGATGTTTCACTAGTTAGGTTAACCCCTACACCAGTCTCCATCATTGCAGGA

**TM9 (454 bp)**

ACATACTCTGATGTTTCACTAGTTAGGTTAACCCCTACACCAGTCTCCATCATTGCAGGAGACAGCCCACATGTTTTGGTCGCATTGTCAAGGCACACCTGTTCGCTCAAGTACTACACTGTTGTTATGGACCCTTTAGTTAGTATCATTAGAGATCTTGAGAAACTTAGCTCGTACTTGTTAGATATGTATAAGGTCGATGCAGGAACACAATAGCAATTACAGATTGACTCGGTGTTCAAAGGTTCCAATCTTTTTGTTGCAGCGCCAAAGACTGGTGATATTTCTGATATGCAGTTTTACTATGATAAGTGTCTCCCAGGCAACAGCACCATGATGAATAATTTTGATGCTGTTACCATGAGGTTGACTGACATTTCATTGAATGTCAAAGATTGCATATTGGATATGTCTAAGTCTGTTGCTGCGCCTAAGGATCAAATCAAACCACTAA

**TM10 (454 bp)**

CTGCGCCTAAGGATCAAATCAAACCACTAATACCTATGGTACGAACGGCGGCAGAAATGCCACGCCAGACTGGACTATTGGAAAATTTAGTGGCGATGATTAAAAGGAACTTTAACGCACCCGAGTTGTCTGGCATCATTGATATTGAAAATACTGCATCTTTAGTTGTAGATAAGTTTTTTGATAGTTATTTGCTTAAAGAAAAAAGAAAACCAAATAAAAATGTTTCTTTGTTCAGTAGAGAGTCTCTCAATAGATGGTTAGAAAAGCAGGAACAGGTAACAATAGGCCAGCTCGCAGATTTTGATTTTGTAGATTTGCCAGCAGTTGATCAGTACAGACACATGATTAAAGCACAACCCAAGCAAAAATTGGACACTTCAATCCAAACGGAGTACCCGGCTTTGCAGACGATTGTGTACCATTCAAAAAAGATCAATGCAATATTTGGCCC

**TM11 (454 bp)**

GTACCCGGCTTTGCAGACGATTGTGTACCATTCAAAAAAGATCAATGCAATATTTGGCCCGTTGTTTAGTGAGCTTACTAGGCAATTACTGGACAGTGTTGATTCGAGCAGATTTTTGTTTTTCACAAGAAAGACACCAGCGCAGATTGAGGATTTCTTCGGAGATCTCGACAGTCATGTGCCGATGGATGTCTTGGAGCTGGATATATCAAAATACGACAAATCTCAGAATGAATTTCACTGTGCAGTAGAATACGAGATCTGGCGAAGATTGGGTTTTGAAGACTTCTTGGGAGAAGTTTGGAAACAAGGGCATAGAAAGACCACCCTCAAGGATTATACCGCAGGTATAAAAACTTGCATCTGGTATCAAAGAAAGAGCGGGGACGTTACGACGTTCATTGGAAACACTGTGATCATTGCTGCATGTTTGGCCTCGATGCTTCCGATGGAG

**TM12 (454 bp)**

GCATGTTTGGCCTCGATGCTTCCGATGGAGAAAATAATCAAAGGAGCCTTTTGCGGTGATGATAGTCTGCTGTACTTTCCAAAGGGTTGTGAGTTTCCGGATGTGCAACACTCCGCGAATCTTATGTGGAATTTTGAAGCAAAACTGTTTAAAAAACAGTATGGATACTTTTGCGGAAGATATGTAATACATCACGACAGAGGATGCATTGTGTATTACGATCCCCTAAAGTTGATCTCGAAACTTGGTGCTAAACACATCAAGGATTGGGAACACTTGGAGGAGTTCAGAAGGTCTCTTTGTGATGTTGCTGTTTCGTTGAACAATTGTGCGTATTACACACAGTTGGACGACGCTGTATGGGAGGTTCATAAGACCGCCCCTCCAGGTTCGTTTGTTTATAAAAGTCTGGTGAAGTATTTGTCTGATAAAGTTCTTTTTAGAAGTTTGTTTA

**TM13-14 (454 bp)**

TTGTTTATAAAAGTCTGGTGAAGTATTTGTCTGATAAAGTTCTTTTTAGAAGTTTGTTTATAGACGGCTCTAGTTGTTAAAGACGTCCTTGCAAAGTTTCGATGGCGCGCCAACCGGAAAAAAGAGTGATGTCCGCAAAGGGAAAAATAGTAGTAATGATCGGTCAGTGCCGAACAAGAACTATAGAAATGTTAAGGATTTTGGAGGAATGAGTTTTAAAAAGAATAATTTAATCGATGATGATTCGGAGGCTACTGTCGCCGAATCGGATTCGTTTTAAATAAGTCTTACAGTATCACTACTCCATCTCAGTTCGTGTTCTTGTCATCAGACCTAGGTCACCCAATAGAGTTAATTGCGGCCGCACGACTGCCGAAACGTTAGATGCTACTCGTAGAGTAGACGACGCAACGGTGGCCATAAGGAGCGCGATAAATAATTTAATAGTAGAATT

**TM15 (512 bp)**

GAAACGTTAGATGCTACTCGTAGAGTAGACGACGCAACGGTGGCCATAAGGAGCGCGATAAATAATTTAATAGTAGAATTGATCAGAGGAACCGGATCTTATAATCGGAGCTCTTTCGAGAGCTCTTCTGGTTTGGTTTGGACCTCTGGTCCTGCAACTTGAGGTAGTCAAGATGCATAATAAATAACGGATTGTGTCCGTAATCACACGTGGTGCGTACGATAACGCATAGTGTTTTTCCCTCCACTTAAATCGAAGGGTTGTGTCTTGGATCGCGCGGGTCAAATGTATATGGTTCATATACATCCGCAGGCACGTAATAAAGCGAGGGGTTCGAATCCCCCCGTTACCCCCGGTAGGGGCCCAGGCCGGCATGGTCCCAGCCTCCTCGCTGGCGCCGGCTGGGCAACATTCCGAGGGGACCGTCCCCTCGGTAATGGCGAATGGGACCCTCTAGAGGGCCGCATCATGTAATTAGTTATGTCACGCTTACATTCACGCCCTCCCCCCAC

**mUkG1 (717 bp)**

ATGGTCAGTGTCATCAAAGAAGAAATGAAGATCAAGTTGCACATGGAAGGTAACGTTAATGGTCATGCCTTTGTTATTGAAGGTGATGGTAAAGGTAAACCATACGATGGTACTCAAACTTTGAACTTGACTGTCAAAGAAGGTGCTCCATTGCCATTCTCTTACGATATTTTGACTAACGCCTTCCAATACGGTAATAGAGCTTTTACTAAGTACCCAGCCGATATCCCAGATTACTTTAAGCAAACTTTTCCAGAAGGTTACTCCTGGGAAAGAACTATGTCTTACGAAGATAACGCTATCTGCAACGTCAGATCCGAAATTTCTATGGAAGGTGATTGCTTCATCTACAAGATCAGATTCGATGGTAAGAACTTTCCACCAAATGGTCCAGTCATGCAAAAAAAGACTTTGAAGTGGGAACCATCCACCGAAATGATGTATGTTAGAGATGGTTTCTTGATGGGTGATGTCAATATGGCTTTGTTGTTGGAAGGTGGTGGTCATCATAGATGTGATTTCAAGACTTCTTACAAGGCCAAGAAGGTTGTTCAATTGCCAGATGCTCATAAGATCGATCACAGAATCGAAATCTTGTCCCACGATAGAGATTACTCCAAGGTTAAGTTGTACGAAAACGCTGTTGCTAGAAACTCTTTGTTGCCATCTCAAGCTTCTAAGTAAGGCGCGCCAACCGGAAAAAAGAGTGATGTCCGC
