# Supplementary material for: Detection of bacterial gene expression elements on Tobacco mosaic virus RNA using cDNA analysis
Source: PLoS One. 2026 Sep 15;21(9):e0358128. doi: 10.1371/journal.pone.0358128 (PMC13577412; doi:10.1371/journal.pone.0358128)
Supplement: Supplementary file 3 [file pone.0358128.s007.docx]

**S2 Table. Primer list**

| **Primers for vector construction** | |
| --- | --- |
| TM1-F | GGAACAAAAGCTGGCTAGAACTAG |
| TM2-R | GATTTCGTCCAAATTGACGTATGAATC |
| TM3-F | GCTTTCCACTTCTCTGAGAACC |
| TM4-R | ACCCTGATCTCTAATGCGTCG |
| TM5-F | AGAGAGGCTCTTGAACAGGAAAC |
| TM6-R | TTCTCCAATCATCGCATGTCAC |
| TM7-F | ATCATGTGGCGCTTTTGGAATATG |
| TM8-R | TCCTGCAATGATGGAGACTGG |
| TM9-F | ACATACTCTGATGTTTCACTAGTTAG |
| TM10-R | GGGCCAAATATTGCATTGATCT |
| TM11-F | GTACCCGGCTTTGCAGACGA |
| TM12-R | TAAACAAACTTCTAAAAAGAACTTTATCAG |
| TM14-R | TTATCGCGCTCCTTATGGCCAC |
| mUkG1-F | ATGGTCAGTGTCATCAAAGAAGAAATG |
| *Pvu*II-forward | TCGCTATTACGCCAGAGTGAATTCGAGCTCGGTACCC |
| *Pvu*II-revers | GATTCATTAATGCAGTTGCATGCCTGCAGGTCGAC |
| TM_2791_-F | GGTTGATGTTGCATACTGGTTGTG |
| TM8-F | TTGTGTTAATTTTCTTGTGGCGATG |
| TM_2828_-F | GGCGATGTCATTGTGCGAAATTG |
| TM_4341_-F | AAGACCACCCTCAAGGATTATACCG |
| TM_4759_-F | TTTCGTTGAACAATTGTGCGTATTACAC |
| SD-mUkG1-F | AACTTTAAGAAGGAGATATACATATGGTCAGTG |
| TM_2809_-F | GTTGTGTTAATTTTCTTGTGGCGATG |
| TM_4758_-F | GTTTCGTTGAACAATTGTGCGTATTACAC |
| **Primers for 5’-RACE** | |
| p_mUkG1 | TTTACCATCACC (5'end-phosphorylated) |
| p_TM8 | TGCGAAACAGAC (5'end-phosphorylated) |
| S1_mUkG1 | GATCAAGTTGCACATGGAAGGTAACG (for 1st-PCR) |
| A1_mUkG1 | TGAACCTCCCATACAGCGTC (for 1st-PCR) |
| S2_mUkG1 | GGAAGGTAACGTTAATGGTCATGCC (for 2nd-PCR) |
| A2_mUkG1 | CGTCGTCCAACTGTGTGTAA (for 2nd-PCR) |
| S1_TM8 | CGATGTCACACATTATCTGAACAGGAG (for 1st-PCR) |
| A1_TM8 | AGAGTAGTTCTGCGTGTCTCCAC (for 1st-PCR) |
| S2_TM8 | GCTTTGTCATGAGCACTTCTTCGG (for 2nd-PCR) |
| A2_TM8 | GGGTACGGGAATCCTGAAACTC (for 2nd-PCR) |
| **Primers for RT-qPCR** | |
| q_cysG-F | TTGTCGGCGGTGGTGATGTC |
| q_cysG-R | ATGCGGTGAACTGTGGAATAAACG |
| q_mUkG1-F | GGTGCTCCATTGCCATTCTCTTAC |
| q_mUkG1-R | CTGGGATATCGGCTGGGTACTTAG |
