# Supplementary material for: Detection of bacterial gene expression elements on Tobacco mosaic virus RNA using cDNA analysis
Source: PLoS One. 2026 Sep 15;21(9):e0358128. doi: 10.1371/journal.pone.0358128 (PMC13577412; doi:10.1371/journal.pone.0358128)
Supplement: Supplementary file 4 [file pone.0358128.s008.docx]

**S3 Table. Promoter sequences**

| T7 promoter | TAATACGACTCACTATAG |
| --- | --- |
